# Molecular and mechanical insights into gecko seta adhesion: multiscale simulations combining molecular dynamics and the finite element method

**DOI:** 10.3762/bjnano.16.141

**Published:** 2025-11-14

**Authors:** Yash Jain, Saeed Norouzi, Tobias Materzok, Stanislav N Gorb, Florian Müller-Plathe

**Affiliations:** 1 Eduard-Zintl-Institut für Anorganische und Physikalische Chemie, Technische Universität Darmstadt, Peter-Grünberg-Str. 8, D-64287 Darmstadt, Germany; 2 Zoological Institute Functional Morphology and Biomechanics, Kiel University, Am Botanischen Garten 1–9, D-24118 Kiel, Germany

**Keywords:** finite element method, gecko adhesion, hybrid modeling, molecular dynamics, multiscale simulations, seta, spatula

## Abstract

Gecko adhesion, enabled by micro- and nanoscale structures known as setae and spatulae, has prompted extensive research. We present a concurrent multiscale computational model of a seta that integrates molecular dynamics to capture molecular interactions at the spatula–substrate interface and finite element method to simulate the mechanical behavior of the larger setal shaft. This hybrid approach enables synchronized simulations that resolve both fine-scale interfacial dynamics and overall structural deformation. The model reproduces key aspects of spatula behavior during adhesion and detachment, showing that spatula–substrate contact evolves through a combination of bending, sliding, and peeling, depending on the spatula’s initial orientation. Our results further demonstrate that lateral sliding can delay detachment, thereby enhancing adhesion strength. The computed pull-off forces and observed mechanisms are consistent with atomic force microscopy measurements and previous simulations. These results align with existing experimental and computational studies. They also overcome scale and resolution limitations inherent in single-scale models.

## Introduction

Geckos possess the ability to adhere to a variety of substrates, a trait attributed to specialized micro- and nanoscale structures on their feet [1–2]. This bioadhesion mechanism has been studied extensively, especially for biomimetic adhesive applications [3–9]. Understanding these interactions presents a formidable challenge in biophysics and materials science due to the extremely small length and time scales involved.

In previous research, we used molecular dynamics simulations to explore various aspects of gecko adhesion [10–13]. We found that humidity increases the force required to pull a spatula off from a substrate [10,12], a phenomenon also observed in high-humidity atomic force microscopy (AFM) experiments. Our investigation into how gecko keratin interacts with hydrophilic and hydrophobic substrates [12] supported the importance of the water-mediating effect [10] and elucidated mechanistic differences depending on surface chemistry. A particle-based mesoscale model of a single gecko spatula was then used to bridge molecular interactions and macroscopic adhesion behavior. This mesoscale model accurately simulated spatula detachment from various substrates and matched experimental pull-off forces observed in AFM studies and pull-off pressures from united atom simulations of gecko keratin [11]. It also helped clarify other aspects of humidity-enhanced adhesion, revealing that keratin softening due to water uptake accounts for only a minor part of the increase in adhesion on very smooth surfaces [13]. The present paper builds on our previous research and extends the treatment to an entire seta with multiple spatulae.

Since purely particle-based simulation techniques are limited by the number of atoms (or coarse-grained beads), it becomes computationally unfeasible to model an entire seta at molecular resolution. A back-of-the-envelope calculation gives an estimate of around 10^14^ atoms for a single gecko seta. Coarse-graining [14–15] can improve the situation, but not by more than 1–2 orders of magnitude. Consequently, multiscale approaches combining continuum methods like the finite element method (FEM) [16–18] with particle-based molecular dynamics (MD) [19–20] are necessary to capture both large-scale geometry and local molecular interactions at the adhesive interface. There have been several pure finite element studies of gecko adhesion, which examine mechanisms like peeling from and pushing (loading) onto a substrate [21–28]. While there have been a few sequential multiscale studies [29–31], there have been no concurrent simulations in which micrometer-scale seta mechanics and molecular spatula–substrate interactions feed into each other at runtime. This work concurrently couples MD and FEM in a single simulation to model multiple spatulae in parallel, all connected to a larger setal shaft. Forces and displacements are exchanged at each iteration, ensuring that spatula-level phenomena (e.g., van der Waals contact, slight sliding at the substrate, and spatula bending) feed back into the global deformation. The continuum and particle domains exchange information through a handshake region using our coupling algorithm, which will be detailed in Section “Methods”. The simulations presented in this paper visualize the mechanical response of whole seta and the peeling of spatulae during pull-off from a dry, smooth, and hydrophobic substrate at a loading rate of 1.88 × 10^12^ pN/s. We also examined adhesion forces and quantified spatula–substrate contacts, while comparing with qualitative and quantitative experimental results where available.

We synchronously simulated 16 spatula–substrate contact sites via molecular dynamics, each coupled through its own bridging domain to a single seta described by finite elements. In this way, forces were transmitted between different spatulae as they would in real life. At the same time, molecular processes between spatulae and substrate under these forces were covered at molecular resolution. This two-way, concurrent coupling turns adhesion from a prescribed boundary condition into an emergent, geometry- and state-dependent response: Local spatula-level peeling, sliding, and contact reorientation feed back into the global branched seta, which, in turn, reshapes the local interface state at the next iteration. Practically, it exposes cross-scale observables that pure MD or pure FEM cannot provide, that is, redistribution of forces across branches (anchor-point loads), spatula-resolved contact maps and sliding velocities during seta preloading/pull-off, and detachment order as a function of branch position and seta deformation. The simultaneous use of multiple MD regions coupled to an FEM model is new. Although 16 spatulae are fewer than the thousands found on a real gecko seta [3,32–35], this number was chosen to align with Sauer and colleagues [22]. Scaling to more spatula–substrate sites is straightforward but would increase runtime.

## Models

### Multiscale seta–spatula model

**Figure 1:**
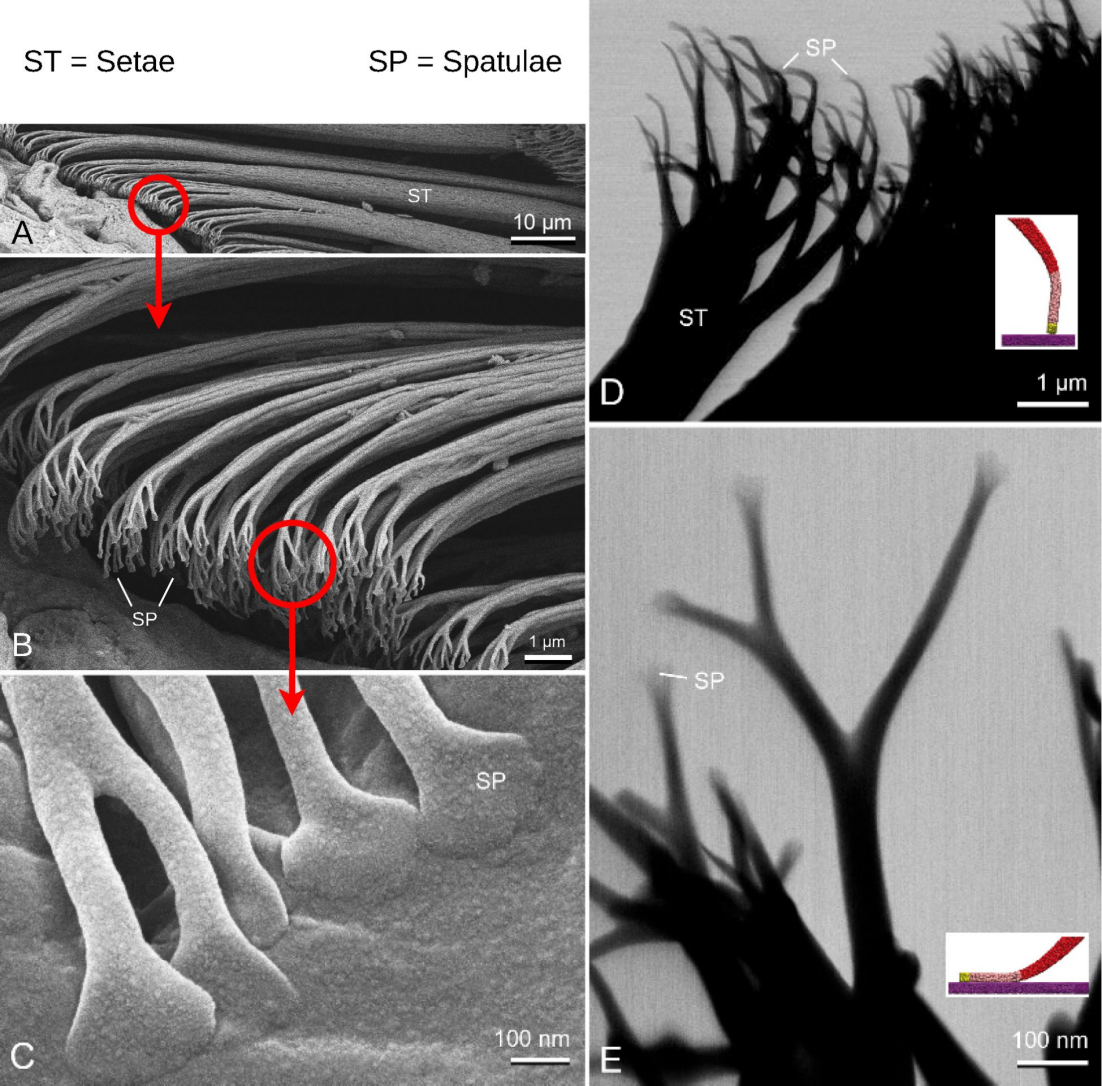
Electron microscopy images of the gecko setae. (A–C) Three hierarchical levels in the scanning electron microscope: A, setae; B, branches of setae (setulae) with the spatulae at the tips; C, spatulae in contact with the surface. The red circles highlight a region while the corresponding arrow points towards a higher resolution (zoomed-in) image of a similar region. (D, E) Setae in contact with the surface with different orientations in the transmission electron microscope. The inset images show corresponding orientations of the spatula molecular model.

**Figure 2:**
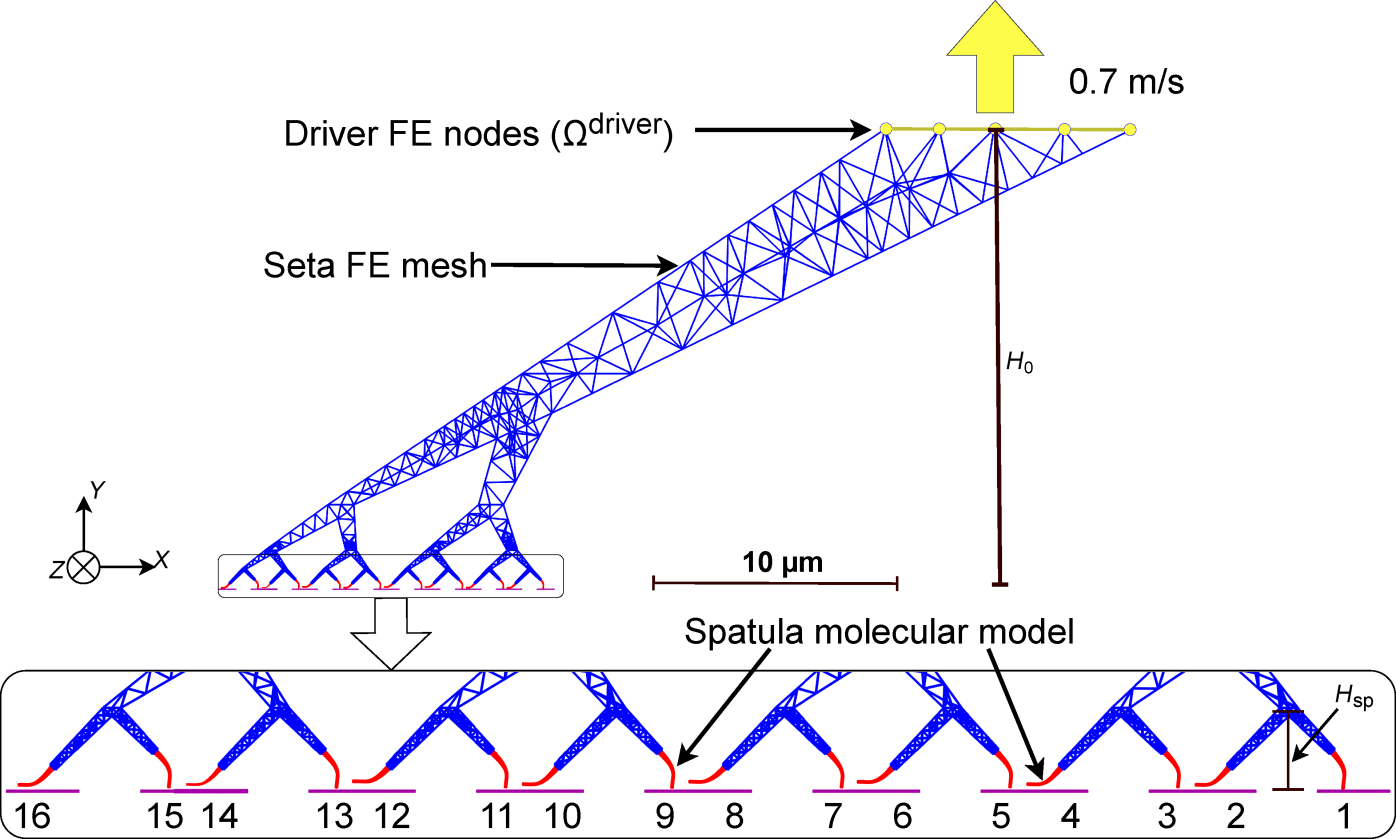
Illustration of the entire multiscale seta model. The red spatulae are modeled using beads, while the rest of the larger blue seta is modeled using a finite element mesh. The driver FE nodes (Ω^driver^) at the top of the seta are subject to an incremental displacement of 1 nm per load step for more than 500 load steps, or until all spatulae are completely detached from the substrate. *H*_sp_ is the height of the spatula including both the final FE branch and the spatula molecular model; *H*_0_ is the total height of the multiscale seta.

A seta branches into spatulae as seen in scanning electron microscopy (SEM) images of gecko setae (Figure 1). A single seta on a gecko’s foot can have dozens of sub-branches, which further branch into hundreds to thousands of spatulae [32–35]. Our seta geometry (Figure 2) was inspired by the branching geometry in Sauer et al. [21], which simplified the seta into distinct branching levels. While this captured key structural features, it did not fully represent the intricate natural hierarchy of real gecko setae, which includes finer branches, varying cross sections, and region-specific material anisotropy. Real setae possess complex cross-sectional shapes and non-uniform branching angles, which could lead to variations in force distributions. Nonetheless, this idealized geometry was sufficient as a first step in validating our concurrent MD–FEM approach and extracting fundamental insights about contact formation, load transfer, and spatula detachment mechanisms.

Simulating each spatula in its own MD simulation, while coupling them all with the seta FEM model, becomes computationally expensive as the number of spatulae increases. Therefore, we limited the model to 16 spatulae per seta. The model can be modified to accommodate different seta morphologies with more spatulae if needed. Figure 2 shows how the setal shaft was simulated with FEM, whereas the spatulae in alternating orientations with their substrates were simulated in 16 parallel MD calculations.

**Figure 3:**
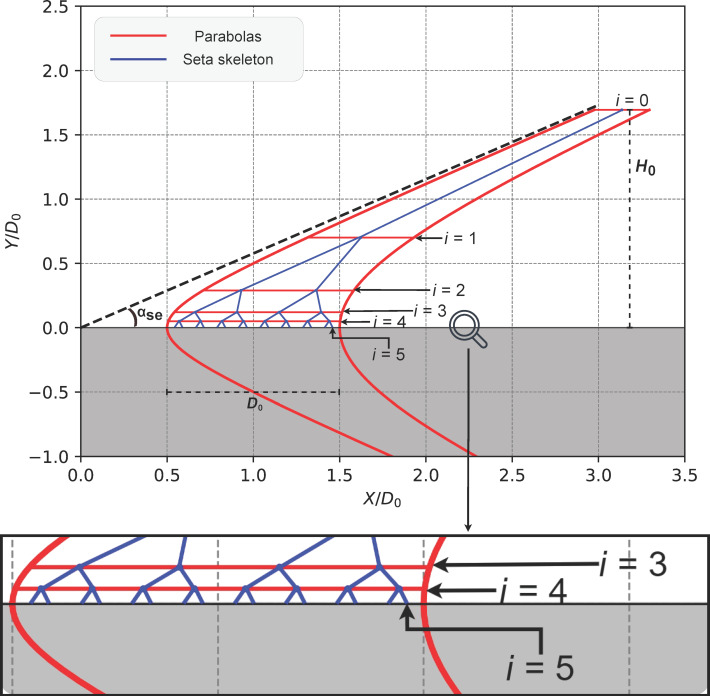
Schematic for constructing the skeleton of our multiscale model. The dimensions of the figure have been normalized with respect to the seta base width *D*_0_ = 13.5 μm. The red lines are the two guiding parabolas that helped construct the seta’s skeleton (blue). A magnified image of the portion with branching points *i* = 3,4,5 shows the final 16 branches that represent our spatulae.

The skeleton of our multiscale seta was created by distributing branching points along two parabolas (Figure 3) as described by Sauer and colleagues [21]. Figure 3 illustrates the construction of the seta FE mesh. The seta was modeled with a height *H*_0_ of 22.8 μm and an inclination angle α_se_ of 30°. The multiscale seta was constructed by modeling a main shaft that branched four times into 16 tips, each connecting to a molecular spatula. The overall seta geometry was bound by the positive legs of the two parabolas (red lines, Figure 3) given by Equation 1 [21],


[1] $$\frac{x^{2}}{C_{1}^{2}}-\frac{y^{2}}{C_{2}^{2}}=1,\;x,y>0,$$

where *C*_1_ = 0.5*D*_0_ and 1.5*D*_0_ for the two boundary parabolas, and *C*_2_ = *C*_1_tan(α_se_). The horizontal line segment between the parabolas at each branching height *H_i_* (where *i* = 1,2,3,4) was divided into 2*^i^*^−1^ equidistant intervals, and the *x*-coordinate of each branching point was the midpoint of one of these intervals. The height of each branching point, starting from the first (highest) branching level, decreased progressively according to Equation 2:


[2] $$H_{i}=\eta^{4-i}H_{\text{sp}},\;i=0,1,2,3,4,$$

where *i* represents the branching level, *H*_sp_ is the total spatula height, including both the final tiny FE branch that connects to the molecular spatula and the molecular spatula itself (see Figure 2), and η is a scaling parameter. The bottom tips of the final branches are at *i* = 5. Joining these branching points with lines formed the skeleton of the multiscale seta (blue lines, Figure 3).

### Seta: finite elements

**Figure 4:**
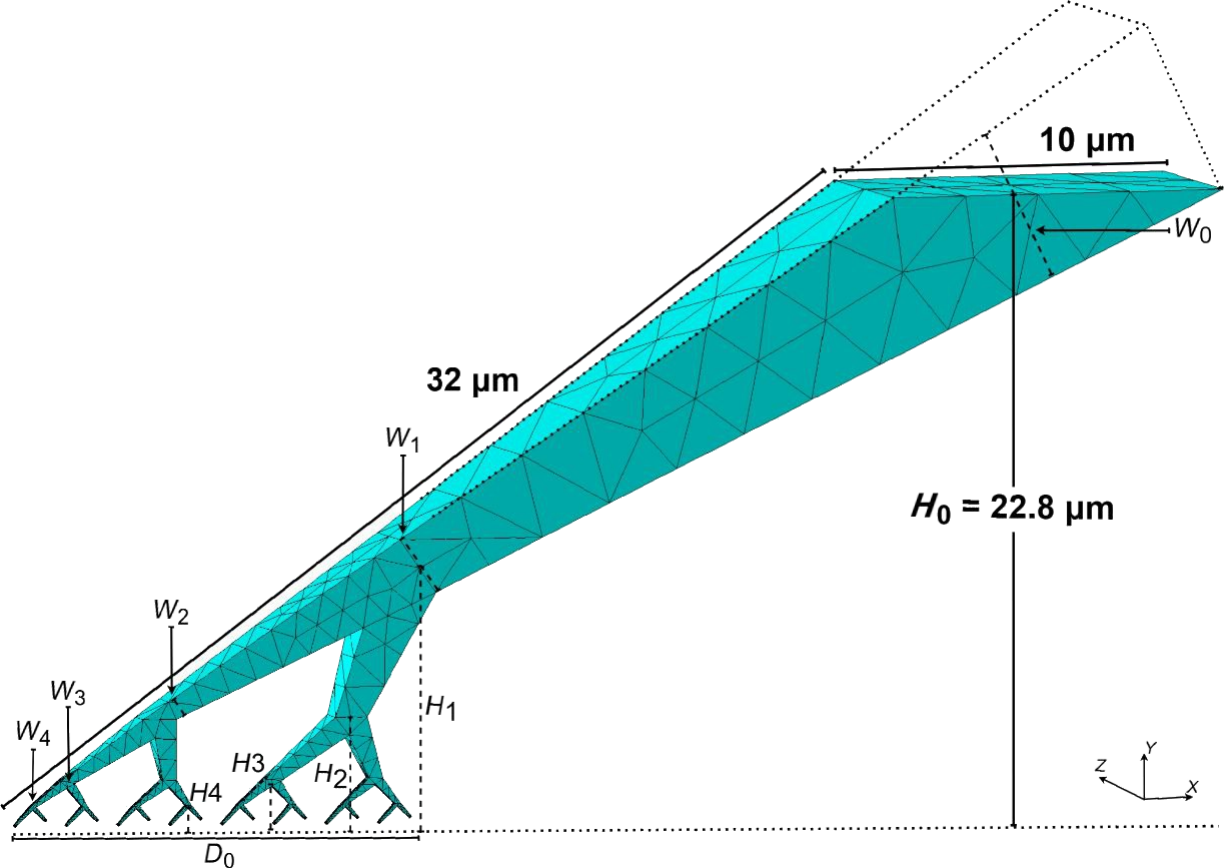
The finite element mesh of the setal shaft in our model. The dotted lines at the top of the seta show the portion that was removed above a height (*H*_0_) of 22.8 μm. Portions of the final tiny FE branches (*i* = 5) were also removed to enable connections to molecular spatulae (here hidden for clarity). A horizontal dotted line shows where the missing portion of the final branches would be, and serves as the reference for displaying heights *H_i_*.

From the seta skeleton, we constructed a three-dimensional finite element mesh using C3D10 tetrahedral elements [36]. The seta’s cross-sectional width *W_i_* (see Figure 4) was determined based on the molecular spatula shaft diameter *R*_sp_ and a scaling parameter γ as given by Equation 3:


[3] $$W_{i}=\gamma^{\left(5-i\right)}R_{\text{sp}},\;i=0,1,2,3,4,5.$$

After the mesh was constructed, the top portion of the seta above a height (*H*_0_) of 22.8 μm was removed. The 45 FE nodes on this cross-sectional area received the prescribed displacement for preloading and pull-off during simulations. They effectively drove the simulation and are referred to as driver FE nodes. Portions of the mesh at the end of the spatulae were also removed to be replaced by molecular spatulae models. Table 1 lists the parameters and values used to construct the continuum part of the model.

**Table 1:** Geometric parameters for the finite element portion of the seta.

Parameter	Value

*H*_0_	22.8 μm
*D*_0_	13.5 μm
α_se_	30°
*R*_sp_	50 nm
η	2.417
γ	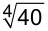
*H*_sp_	0.67 μm

The finite element mesh response was governed by linear isotropic elasticity, with the stress–strain relationship given by Hooke’s law [36–37]:


[4] σ=λtr(ε)I+2με,

where **σ** is the Cauchy stress tensor, **ε** is the infinitesimal strain tensor, tr(**ε**) denotes the trace of the strain tensor, **I** is the second-order identity tensor, and λ and μ are the Lamé parameters defined as:


[5] $$\lambda =\frac{E\nu }{\left(1+\nu \right)\left(1-2\nu \right)}$$

[6] $$\mu =\frac{E}{2\left(1+\nu \right)}$$

with *E* being Young’s modulus and ν Poisson’s ratio.

The real seta material may exhibit anisotropy and viscoelastic or even plastic behavior, especially under rapid loading. Our FE mesh is presently limited to a linear-elastic, isotropic constitutive law, with parameters *E* and ν matching those of our molecular keratin spatula model [11–12]. These values were derived in a bottom-up manner from atomistic simulations that reproduced experimental data [8,38–39]. For each of our simulations, we sampled *E* and ν from normal distributions with the same mean and standard deviations as our mesoscale spatula model. Table 2 summarizes the material parameters used for our FE mesh.

**Table 2:** Mechanical parameters for the finite element portion of the seta model. ^a^

Parameter	Value

constitutive law	isotropic linear elasticity
Young’s modulus (*E*)	4.518 ± 0.036 GPa
Poisson’s ratio (ν)	0.401 ± 0.002

^a^For every simulation, we sampled Young’s modulus and Poisson’s ratio from normal distributions with these means and standard deviations.

### Spatulae and substrate: particles

#### Mesoscale spatula model

**Figure 5:**
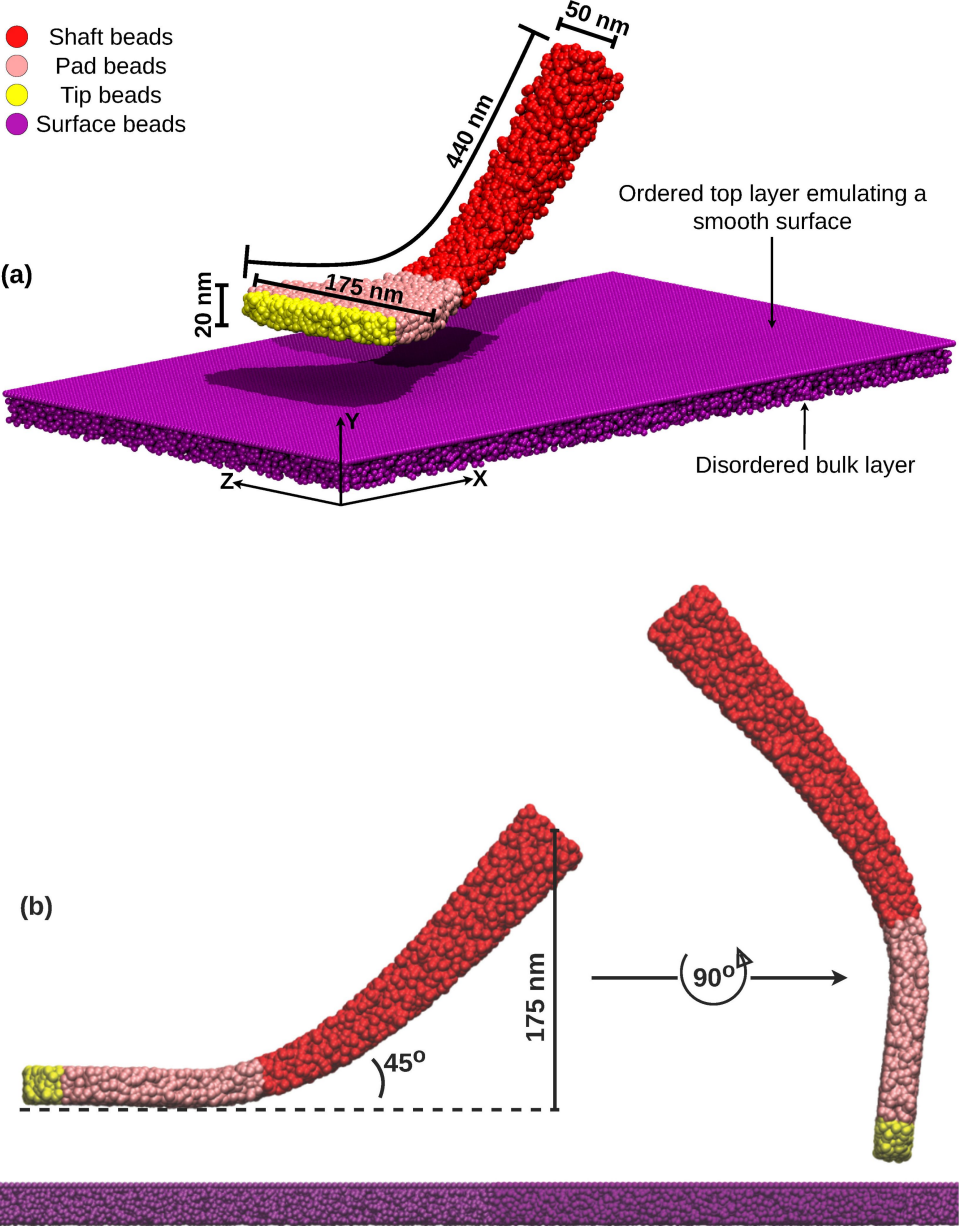
Geometry of the molecular spatula and substrate. (a) A spatula interacting with the dual-layered smooth substrate and (b) the two orthogonal orientations of the spatula in our seta model.

The mesoscale spatula model was derived from prior research. Its shape is based on SEM images [23], and the force field was derived bottom-up from united-atom gecko keratin simulations [12]. The keratin proteins in gecko seta and spatulae form a structure similar to a fiber-reinforced elastomer, with a fibrillar nature that leads to anisotropic mechanical properties [40–42]. Rather than explicitly resolving individual fibers and the surrounding amorphous matrix, we represented the spatula with a coarse-grained bead network whose anisotropic fibrillar structure was encoded through direction-dependent bond stiffnesses. Figure 5a shows the molecular spatula with beads colored according to different regions (tip/pad/shaft). Each bead, representing approximately five keratin molecules or 2.5 keratin dimers (65228 Da), was connected to 30 neighboring beads via harmonic bonds,


[7] $$V\left(r\right)=\frac{1}{2}K{\left(r-b_{0}\right)}^{2},$$

where *r* is the distance between the two beads, and *b*_0_ is the initial (equilibrium) distance between them. Their force constants *K* increased when the bonds aligned with the local keratin fibril direction,


[8] $$K=k+k_{\text{b}}\cdot \left|\cos\theta \right|,$$

where *k* is the base isotropic force constant identical for all bonds, *k*_b_ is an additional force constant in the fibril direction, and θ is the angle between the bond vector and the fibril direction. We systematically tuned the parameters *k* and *k*_b_ so that the resulting coarse-grained network reproduced the spatula’s key mechanical properties, specifically, its Young’s modulus and Poisson’s ratio, as derived from atomistic simulations and experimental data [11]. We generated ten such spatula models with differing bead positions and bond constants, yielding a Young’s modulus of 4.518 ± 0.036 GPa and a Poisson’s ratio of 0.401 ± 0.002. Details can be found in our previous work [11]. These spatula models were coupled to the FE seta in two orthogonal orientations as shown in Figure 5 and Figure 2b.

#### Smooth substrate

We employed a simple particle-based model of a nonpolar (hydrophobic), smooth substrate as a baseline to remain consistent with the previously validated parametrization of our coarse-grained potential against united-atom simulations of gecko keratin on nonpolar surfaces [10]. Hydrophilic substrates, roughness, and humidity, which introduce additional mechanisms such as keratin softening and capillary forces, have been addressed in our previous spatula-scale studies [12–13] and will be treated in future multiscale work. In our previous work [10], we only investigated spatula detachment in a direction normal to the substrate, so a fully random bead arrangement posed no risk of the spatula beads sliding into frozen/immobile substrate pores. In the present simulations, the spatula was free to slide laterally, which could have risked “locking” if the substrate was entirely disordered, contained random voids, and was inflexible. To prevent this, we introduced a two-layer substrate, that is, a lattice-like monolayer of beads on top, forming a smooth, cavity-free surface, and a 13 nm thick amorphous bulk below, which matched the same bead density as our previous random-substrate model. All substrate beads had identical interactions with the spatula, and both layers were held fixed in space. Since the regular top monolayer differed from the fully random arrangement used previously, we re-parameterized the spatula–substrate Lennard-Jones interactions:


[9]

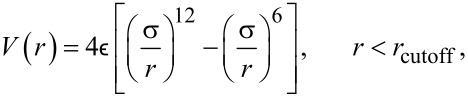



so that the new surface would have the same pull-off (adhesion) pressure as before. Specifically, several parameter combinations were tested in pull-off simulations against our reference data for gecko keratin [12], and the parameter set that reproduced the target adhesion (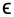 = 290 kJ/mol, σ = 4 nm, and *r*_cutoff_ = 12 nm) was selected. The relatively large σ value reflects the coarse graining, and *r*_cutoff_ = 3σ retains most of the attractive tail of the LJ potential. Any surface bead deeper than the cutoff (12 nm) does not interact with the spatula beads; therefore, any substrate thicker than the spatula–substrate potential cutoff (*r*_cutoff_ ≤ thickness ≤ ∞) would result in identical dynamics and forces. Our substrate (1 monolayer + 13 nm amorphous bulk) exceeds the cutoff, fully representing all interactions while avoiding unnecessary computational cost. We note that the substrate model is an idealized approximation chosen for consistency with our prior work [10,12] and computational efficiency. Modeling biologically realistic interfaces will require extending the present model to include stratification, surface roughness, substrate deformability, and explicit long-range electrostatics for hydrophilic systems, which will necessitate thicker substrate models.

The coupling between each molecular spatula and the FE seta was handled via a bridging domain (BD), described in section “Methods”.

## Methods

### Multiscale coupling algorithm

The coupling algorithm connects three distinct domains, each governed by different physical principles and simulation techniques: (1) The particle domain, that is, the set of all particles simulated using molecular dynamics (MD). This domain captures molecular interactions, structures, and processes at the spatula–substrate interface. (2) The continuum domain, that is, the set of all nodes and elements simulated using the finite element method (FEM). This domain models large-scale mechanical behavior. (3) The bridging domain (BD), that is, a handshake region that incorporates elements of both MD and FEM. It facilitates synchronous information exchange between the particle and continuum domains through the coupling algorithm.

**Figure 6:**
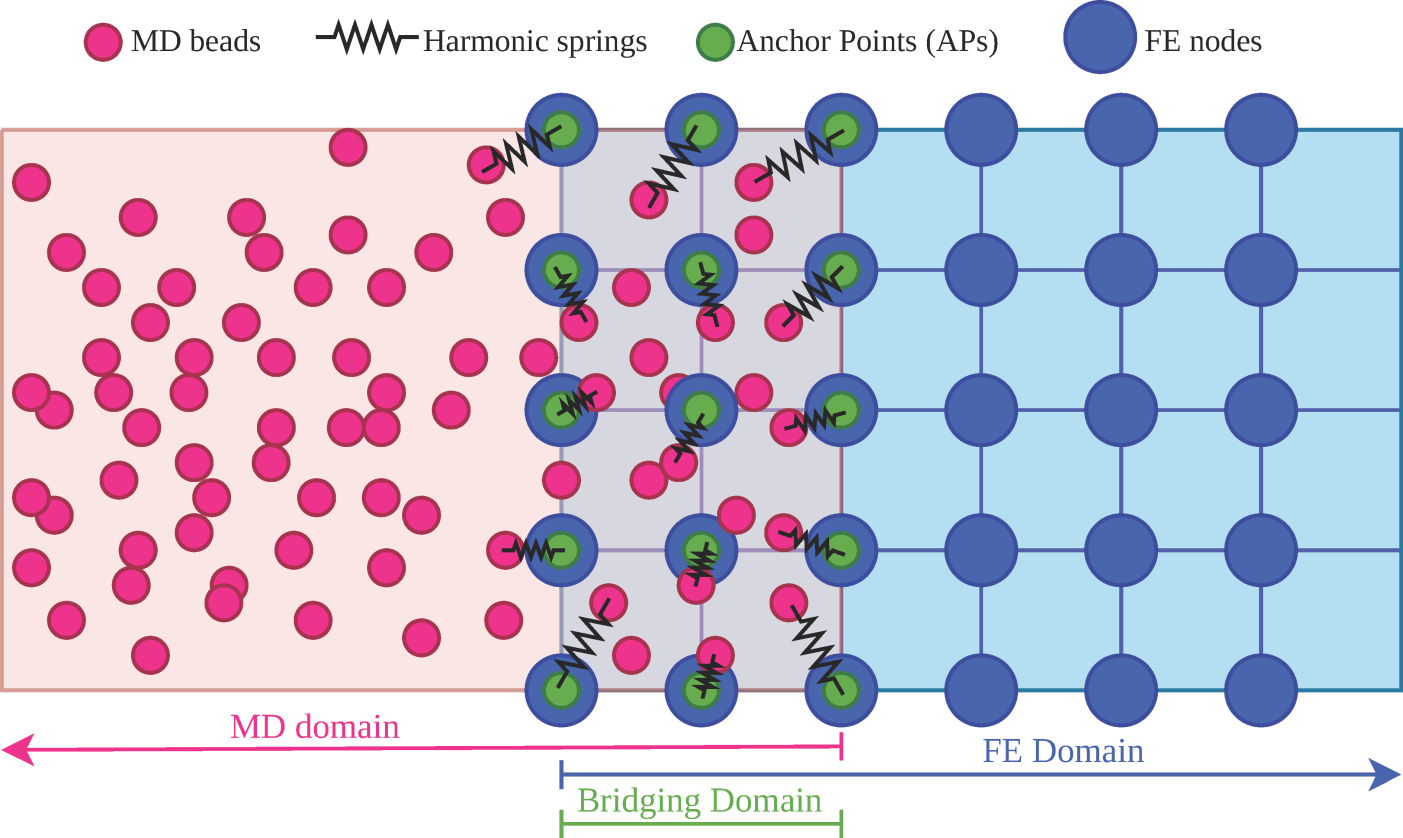
Illustration of the three domains: particle (MD), continuum (FE), and bridging domain (BD). Also shown are the FE nodes, MD beads, anchor points (APs), and harmonic bonds connecting APs to MD beads.

Figure 6 illustrates these three domains. The pink region, consisting of MD particles, is the particle domain; the blue region, composed of a finite element mesh with nodes, is the continuum domain; and the overlapping grey region, which includes coupling components such as anchor points (APs) and harmonic springs, is the bridging domain. Calculations alternate between MD and FEM. The finite element mesh and anchor points (see subsection “Anchor points and force transfer”) are held fixed while MD runs for several hundred picoseconds (140 ps or 7000 time steps in our case). Then, the MD particles are frozen and the FEM is solved. These alternations are continued until the process of interest is complete.

#### Anchor points and force transfer

To connect the particle and continuum domains, APs are placed in the bridging domain. The APs are virtual particles fixed in space that coincide with the positions of FE nodes in the bridging domain. Each AP is connected to a nearby MD bead (not already linked to another AP) via a harmonic spring. The initial AP–MD bead distance serves as the equilibrium length of the harmonic spring. Figure 6 shows the APs as green particles in the bridging domain. Each green AP coincides with a blue FE node and is connected to a pink MD bead by a black harmonic spring. A suitable choice of the MD-coupling spring constant produces forces of the same order of magnitude as the forces among MD beads. In our simulations, the same force constant as for bonds within the molecular model was used. During each MD iteration, forces exerted on the APs by the MD system are recorded, averaged over the final portion of the trajectory (typically 30–50%), and then transferred to the corresponding FE nodes.

#### Controlling FEM deformation

In the FEM calculation, forces transferred through the APs are applied as external loads on the corresponding FE nodes. These loads cause deformation of the FE mesh. However, since they remain constant during each FEM run, the mesh deformation may not fully reflect interactions where forces evolve dynamically based on spatula behavior. In particular, during loading simulations such as multiscale seta pull-off, this can lead to excessive FE mesh deformation due to missing dynamic contributions from the spatulae.

To mitigate excessive deformation or drift, harmonic springs connect the FE nodes in the bridging domain to their initial positions at the start of each FEM iteration. These springs have a stiffness referred to as the FE-coupling spring constant. These FE-coupling harmonic springs are not shown in Figure 6. By providing a restoring force, they ensure controlled deformation of the FEM mesh and prevent overshooting beyond equilibrium. Additional theoretical details on how the FE-coupling springs act as a penalty term in the FEM calculation can be found in our previous work, which used this multiscale approach to simulate crystalline and amorphous polymers [43]. Once the FEM calculation is complete, the updated positions of the FE nodes in the bridging domain, and hence of the APs, are passed back to the particle domain. The FE-coupling spring constant was optimized, starting from the same value as the MD-coupling spring constant and then reduced until forces across the domains matched [43]. Further discussion on the choice of the spring constants is provided in Supporting Information File 1. The FE calculation is static (no inertia), and the MD employs thermostats; accordingly, strict global momentum conservation across both domains is not enforced. Instead, the appropriate consistency criterion is traction–displacement matching at the interface. Traction–displacement consistency refers to enforcing both displacement continuity and traction equilibrium in the bridging domain between a continuum domain and a particle domain. In practice, this means that the FE and MD domains share matching deformations at their boundary (no jump in displacements), and the forces/stresses transmitted across the interface are balanced (no net traction mismatch) [44–47]. The reactions of the FE-coupling springs remain internal to the FE solver; their influence on MD enters only via the updated positions of bridging domain FE nodes, which serve as the new AP positions for the subsequent MD run.

#### Iterative workflow and load steps

After the FE calculation, the MD system evolves under the external potential of the updated AP positions, and the averaged forces on the APs are then calculated and transferred back to the FEM simulation. This cycle of FEM and MD simulations constitutes a single FEM–MD iteration.

Synchronization of the domains requires multiple iterations, not only due to deformation control from the FE-coupling spring but also due to interplay between molecular and continuum forces. The number of required iterations depends on the FE-coupling spring constant and should be considered during their optimization. A smaller FE-coupling spring constant leads to greater mesh deformation and, thus, represents a larger step towards the final FEM solution. This requires fewer iterations but also increases the risk of numerical instabilities. Conversely, a larger spring constant results in smaller steps towards equilibrium but requires more iterations.

External loading (e.g., displacement or force) on the full system, such as moving the entire seta, is applied in increments called load steps. Each load step consists of (1) applying a portion of the total load (a displacement of 1 nm in our case) to the driver nodes and (2) running multiple FEM–MD iterations (ten in our case) under the applied load until the required loading rate is matched (for non-equilibrium simulations), or system energies and AP forces converge (for quasi-static equilibrium simulation).

For instance, a total displacement of 10 nm is applied in ten load steps of 1 nm each, with several FEM–MD iterations at each step. The maximum loading rate that can be imposed and the number of iterations per load step depend on the system size and dynamics. Since the FEM calculation is static and lacks time information, the time corresponding to a load step, Δ*t*^ls^, is estimated using the MD time step, Δ*t*^MD^, the number of MD steps per iteration, *n*^MD^, and the number of iterations per load step, *n*^iter^:


[10] $$\Delta t^{\text{ls}}=n^{\text{iter}}n^{\text{MD}}\Delta t^{\text{MD}}.$$

Using this time and the imposed strain per load step ε^ls^, the pull-off velocity is set as


[11]

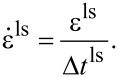



In our previous work [46–50] on bulk polymer models, we found that prescribing a displacement of around 0.004–0.1% of the system size per load step was effective. For our seta model, a 1 nm displacement is 0.004% of the initial height. Furthermore, a parameter study for our polymer models [47] showed that 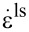 should be small enough (50–100% of a polymer bond length) to avoid a excessively large strain on the MD domain. A displacement of 1 nm corresponded to 33% of the shortest bond (3 nm) and 14% of the average bond length (7.33 ± 2.16 nm) in our molecular spatula model.

In pull-off experiments, the loading rate is defined as the rate at which adhesion forces increase during pull-off. The spatula–substrate MD force field was parametrized to match atomistic keratin pull-off simulations at a loading rate of 1.66 × 10^12^ pN/s [10,12–13], and so we aimed to match the same rate. Preliminary pull-off tests identified a pull-off velocity of 0.7 m/s which yielded a loading rate of 1.88 × 10^12^ pN/s. For a displacement (ε^ls^) of 1 nm and a velocity (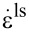) of 0.7 m/s, our load step time (Δ*t*^ls^) then corresponded to 1.4 ns according to Equation 11.

The parameter study for our previous multiscale polymer models [47] showed that such an approach, in general, is robust with respect to the number of iterations per load step (*n*^iter^). This observation holds for our current simulations as well. Different choices of *n*^iter^ between one and ten are explored in Supporting Information File 1. Our choice of *n*^iter^ = 10 is large enough for agreement between pre-detachment forces in different domains, while remaining computationally feasible. If instead a quasi-static equilibrium simulation is desired, then Δ*t*^iter^ and *n*^iter^ must both be high enough (thereby also increasing Δ*t*^ls^ in Equation 10) for the multiscale system to be in equilibrium at the end of every load step.

### Seta–spatula bridging domain

**Figure 7:**
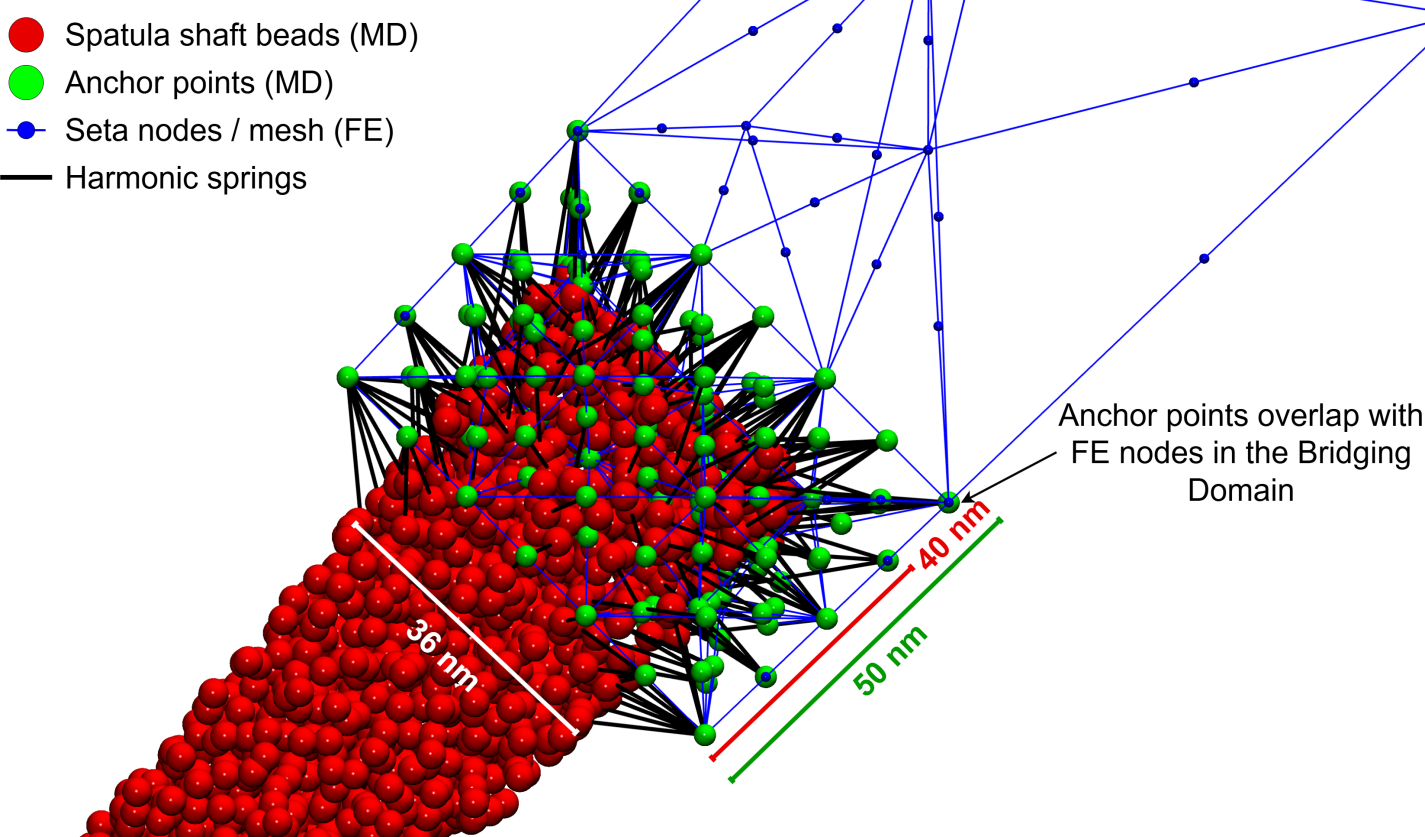
A bridging domain connects one of the spatulae (modeled using molecular dynamics) to the setal shaft (modeled using finite elements).

In the multiscale model, there were 16 bridging domains (BDs), one for each spatula. Each BD needed to be of a size that could be described by both (MD and FEM) methods. The FE portion of each BD was roughly 79 × 75 × 50 nm^3^ in size, and the overlapping MD portion was roughly 40 × 36 × 39 nm^3^ (Figure 7).

The number of APs per BD averaged 395 ± 9.9, depending slightly on the local FE mesh geometry. Each AP coincided with a FE node and was linked to a nearby MD bead via a harmonic spring with stiffness (MD-coupling spring constant) 0.28 nN/nm. FE nodes in the BD were also coupled to their initial positions via springs with stiffness (FE-coupling spring constant) 0.08 nN/nm. Figure 7 illustrates a typical BD configuration, showing the overlapping domains, anchor points, and harmonic spring connections.

## Simulation Details and Parameters

### Iteration details

#### Molecular dynamics

We used the velocity-Verlet algorithm [51] under canonical (NVT) ensemble to perform time integration of the spatula beads and update their positions and velocities. A velocity rescaling thermostat [52] with a coupling time of 2 ps acting only outside the BD maintained the temperature of the spatulae at 300 K, similar to our previous work [11,13]. Since the simulation assumed a frictionless vacuum environment, thermostats like dissipative particle dynamics and Langevin [53–54], which introduce implicit friction, are inherently unsuitable. In particular, Langevin dynamics led to unphysical effects such as loss of spatula jump-to-contact (snap-in) when near the substrate and absence of post-detachment wiggling. Consequently, a weak Berendsen thermostat [55] with a coupling time of 10 ps proved sufficient to maintain the temperature of the bridging domain while dissipating the kinetic energy introduced by load steps. Each AP experienced a force due to its harmonic bond with a connected MD bead. This force was averaged over the final 50% (70 ps) of the MD trajectory (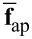) and then applied as an external boundary condition to the next FEM iteration as per Equation 12. The MD iterations were conducted with LAMMPS [56]. Table 3 lists further details and parameters of the MD simulations.

**Table 3:** Molecular dynamics iteration parameters.

Parameter	Value

ensemble	NVT
thermostat outside the bridging domain	velocity rescaling [52]
thermostat inside the bridging domain	Berendsen [55]
thermostat temperature	300 K
velocity rescaling thermostat coupling time	2 ps
Berendsen thermostat coupling time	10 ps
time step	20 fs
non-bonded interaction cutoff	120 Å
total time per iteration	140 ps
force-averaging period	last 70 ps

#### Finite element iterations

In the FEM part, boundary conditions were applied on two distinct node groups, namely, the driver group (Ω^driver^) and the bridging domain (Ω^BD^) group. The 45 driver nodes (Ω^driver^) located on the top end of the seta (Figure 2) were subjected to Dirichlet boundary conditions: In the first iteration of each load step, a prescribed displacement of 1 nm was applied in the *Y*-direction (negative for preload; positive for pull-off), with motion in *X*- and *Z*-directions fully constrained. In all subsequent iterations within the same load step, the driver nodes were fully constrained in all three directions.

The BD nodes (Ω^BD^) were subjected to Neumann boundary conditions, where averaged forces on the anchor points 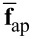 computed from the MD iteration were applied as external forces on the BD nodes:


[12] $$f_{\text{ext}}={\bar{f}}_{\text{ap}}\text{ on }\Omega^{\text{BD}}\text{ (all iterations)}.$$

As discussed in subsection “Controlling FEM deformation”, we attached linear elastic springs to all nodes in Ω^BD^, anchoring them to their initial reference positions. These springs introduced a restoring force proportional to the node displacement **u**:


[13] $$f_{\text{spring}}=-k^{\text{FE}}u\text{ on }\Omega^{\text{BD}},$$

where *k*^FE^ is the FE-coupling spring constant. Therefore, the final external force on these nodes becomes


[14] $$\left(K+K^{\text{FE}}\right)u=f_{\text{ext}}.$$

Here, **K**^FE^ is a diagonal matrix representing spring contributions assembled over Ω^BD^.

The remaining nodes of the finite element mesh adjusted their positions in response to the displacement of the top and the forces transmitted from the molecular dynamics domain at the bottom by solving Equation 14 as prescribed by the constitutive law discussed in subsection “Seta: finite elements”. At the end of the FEM iteration, positions of nodes in Ω^BD^ (**r**_bdfe_) were set as new anchor point positions for the next MD iteration. The FEM iterations were conducted with ABAQUS [36].

### Simulation protocol

We aimed to replicate the protocol used in AFM experiments on gecko spatulae and setae as reported in [23,57]: (1) Initially, all spatulae were positioned at a minimum distance of 13 nm above the substrate, which is higher than the non-bonded interaction cutoff of 12 nm. (2) In the preloading phase, a 1 nm downward displacement towards the substrate was applied to the driver FE nodes at the start of each load step. Each load step consisted of ten FEM–MD iterations. During this phase, the 16 spatulae were gradually pushed onto the substrate by the moving seta. This process continued until a total preload (spatula–substrate) force of 320 nN combined from all spatulae was reached, ensuring that all spatulae established contact with the substrate. This preload force is similar to the average 18–20 nN per spatula imposed in AFM experiments [23,58]. (3) Following preloading, a set of 10 FEM–MD iterations (1.4 ns) was conducted without any applied displacement. Unlike in typical AFM experiments, where the seta is pulled sideways (sheared) to increase the contact area, we did not actively shear. However, spontaneous sliding of the spatulae was observed during preloading. (4) After relaxation, during the pull-off (loading) phase, the seta top was moved upwards by reversing the direction of the applied displacement, again using 1 nm per load step. This eventually led to detachment of the spatulae from the substrate. The pull-off was continued until all spatulae were outside the interaction range of the substrate.

To ensure statistical robustness, we conducted five simulation runs using independently generated multiscale seta models. The same seta FE mesh, with slightly perturbed Young’s modulus and Poisson’s ratio (see subsection “Seta: finite elements”), was attached to 16 spatulae. Each spatula was randomly selected from the set of ten uniquely generated spatula models (see subsection “Spatulae and substrate: particles”).

## Results and Discussion

### Forces, contacts, and displacement profiles

AFM experiments measure forces exerted by the cantilever tip during pull-off, with the maximum pulling force representing the adhesion force between the spatula and the substrate [57]. These experiments typically report the force required to detach a spatula, but they are limited to measuring forces at the cantilever tip and cannot directly resolve molecular-scale interactions at the spatula–substrate interface. In contrast, our hybrid MD–FE model enables us to compute forces at multiple locations throughout the system and to track the dynamics of individual spatulae during preloading and pull-off. To analyze adhesion behavior, we plotted force, contact, and displacement profiles, which provide complementary perspectives on the detachment process.

#### Force profiles

We calculated forces at three points in the system: (1) Spatula–substrate adhesion forces, that is, the total force exerted by the substrate on the spatulae through their interactions. These were averaged over the last 3500 time steps or 70 ps of the final MD iteration of a load step. (2) Anchor point forces, also averaged over the last 3500 time steps or 70 ps of the final MD iteration of a load step. (3) Driver FE node forces, which were not time-averaged and were computed directly at the end of each load step.

In all force profiles presented in this study, compressive forces, where two entities press against each other, are reported as positive, while tensile forces, which represent adhesion during pull-off, are negative. When reporting an “adhesion (pull-off) force” in the text, we quote the magnitude of the minimum force, that is, |*F*_min_|, unless stated otherwise.

#### Contact profiles

We tracked the number of spatula–substrate contact points throughout the simulation. A contact point is defined as the distance between a spatula bead and a substrate bead being less than 12 nm. So long as any spatula bead was interacting with any substrate bead (i.e., any bead–bead distance smaller than the cutoff), the spatula was considered to be in contact with the substrate. The number of such interactions is referred to as the number of contact points. The contact profiles provide a measure of how many spatulae, and through how many contact points, adhered to the substrate at any point in the simulation, helping us understand the detachment sequence and the extent of adhesion loss during pull-off.

#### Displacement profiles

To further investigate how individual spatulae detach, we tracked the displacement of spatula tips and pads over time. The tip refers to the distal end of the spatula, which is typically the last point of contact during detachment, while the pad represents the main surface of interaction between the spatula and the substrate. Figure 5 shows the “tip” and “pad” beads in our spatula models, colored yellow and pink, respectively. We defined the *X*-axis as parallel to the substrate and the *Y*-axis as perpendicular (normal) to it (see Figure 2). The +*X* direction points toward spatula no. 1 in Figure 2 (i.e., along the elevation of the setal shaft), while the +*Y* direction follows the applied pulling force. The displacement profiles for each group of beads (tip or pad) were computed by averaging the positions of all beads in that group. These displacement profiles help distinguish between peeling, shearing, and other modes of detachment, and enable correlating displacement trends with force and contact loss.

### Seta behavior

#### Forces during preloading and pull-off

**Figure 8:**
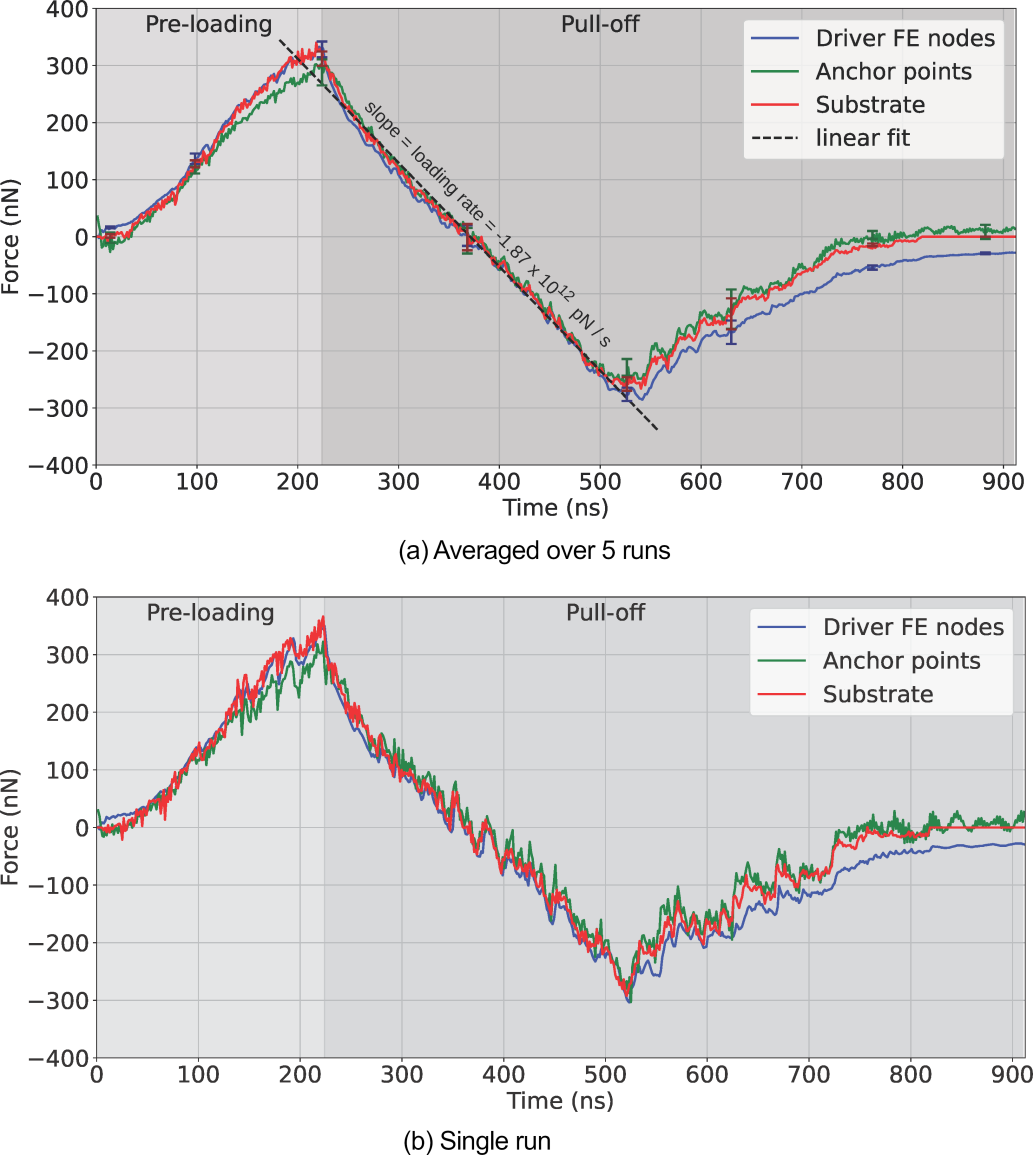
Reaction forces experienced by the substrate (red), anchor points (green), and the driver FE nodes (blue) in the pull-off simulations of entire gecko setae. (a) Averaged over five runs using different realisations of the spatulae. Error bars with darker colors have been plotted at interesting points in the curve. (b) For a single run. By convention, compression during preloading is positive, and adhesion during pull-off is negative; the adhesion peak corresponds to the minimum of the curves.

Once preloading began (Figure 8) and the spatulae made initial contact with the substrate, further load pressed the spatulae against the substrate. The added spatula–substrate forces reached 320 nN after 224 ns (load step 160), marking the end of the preloading phase. In Figure 8, the relaxation phase is the single data point at 225.4 ns (load step 161) consisting of ten FEM–MD iterations (1.4 ns), and the transition to pull-off is identified by a reversal in the force direction from positive (compression) to negative (adhesion). As the pull-off continued, we crossed a point at 364 ns (load step 260) where the seta and substrate remained in contact but the net spatula–substrate force was zero. Beyond this point, the attractive force became more negative, reaching a minimum of −(284.5 ± 14) nN at 526 ns (load step 376). If all 16 spatulae were to detach simultaneously, this would give a naive average spatula adhesion force of ≈18 nN per spatula (reported as |*F*_min_|), an averaging method previously found in AFM experiments [57]. Spatula contact analysis (detailed in the following subsection “Spatula behavior”) revealed that, at this moment, 14 of 16 spatulae were still in contact with the substrate (i.e., only spatulae 1 and 3 had detached) and several pad-dominant spatulae achieved their own force minima later. Consequently, dividing the seta-level minimum by 16 (≈18 nN) underestimates the per-spatula adhesion force. A more accurate calculation involves averaging the force of each individual spatula at its own force minima, which yielded an average adhesion force of |*F*_min_| = 33.2 ± 4.7 nN. This matches the adhesive force of ≈35 nN reported in our previous work for the same molecular spatula model at a similar loading rate [10,13]. In the previous work, we conducted molecular simulations at various loading rates and extrapolated the adhesion forces down to rates which are typical in AFM measurements. At those lower loading rates, we saw extrapolated adhesion forces in the range of 12–25 nN, which matched the range of 8–20 nN observed in AFM experiments [35,57,59]. As detachment progressed, spatulae peeled off one by one, causing a gradual return of the force toward zero. The bumps in this detachment regime of Figure 8b result from a rise in the spatula–substrate adhesion force just before a spatula detaches and then the subsequent return due to detachment. Complete seta detachment occurred when the substrate reaction force dropped to zero due to all spatulae detaching at around 840–860 ns (load steps 600–615).

Despite discrepancies arising from the energy exchange via thermostats in the MD domain and differences in how each force is computed and averaged, the three different forces in Figure 8a remain closely aligned until detachment begins. Beyond this point, the curves begin to diverge. This divergence is not primarily due to the physical mechanics of detachment, but rather due to the limited number of FEM–MD iterations per load step in our runs (i.e., we mimic non-equilibrium AFM pulling), which prevented the system from fully equilibrating after rapid detachment events. Further discussion and simulations showing the effect of varying the number of FEM–MD iterations per load step on the force profiles are presented in Supporting Information File 1.

As individual spatulae detached, some slightly before and many after the seta-level force minima (peak adhesion) at ≈526 ns, they released stored elastic energy and accelerated rapidly. This sudden increase in velocity caused wiggling and rapid configurational changes within the MD domain. Due to the limited number of FEM–MD iterations per load step, the FE mesh did not have sufficient opportunity to adapt to these fast dynamics. As a result, the anchor point and driver FE node forces retained residual mismatches that were not fully dissipated, even after the substrate reaction force (red curve) dropped to zero. In the single-run results of Figure 8b, this manifests itself as persistent fluctuations around non-zero values in the AP and driver FE forces beyond ≈840 ns, when all spatulae are fully detached. These fluctuations arise from post-detachment wiggling of the spatulae. However, the concerning aspect is that these forces oscillate around non-zero values even after the substrate force has vanished. In Supporting Information File 1, we provide additional tests where load steps were extended up to 400 FEM–MD iterations. They confirm that the force divergence seen in the post-detachment phase is significantly reduced, or even eliminated, if sufficient iterations are allowed. However, such high iteration counts are not only computationally unfeasible with our available hardware but also go against a fast loading rate of 10^12^ pN/s. Therefore, while force and contact behaviors before detachment are well resolved and converged, the post-detachment region (after ≈526 ns or load step 376) should be interpreted with caution in terms of exact force magnitudes.

#### Seta–substrate contacts

**Figure 9:**
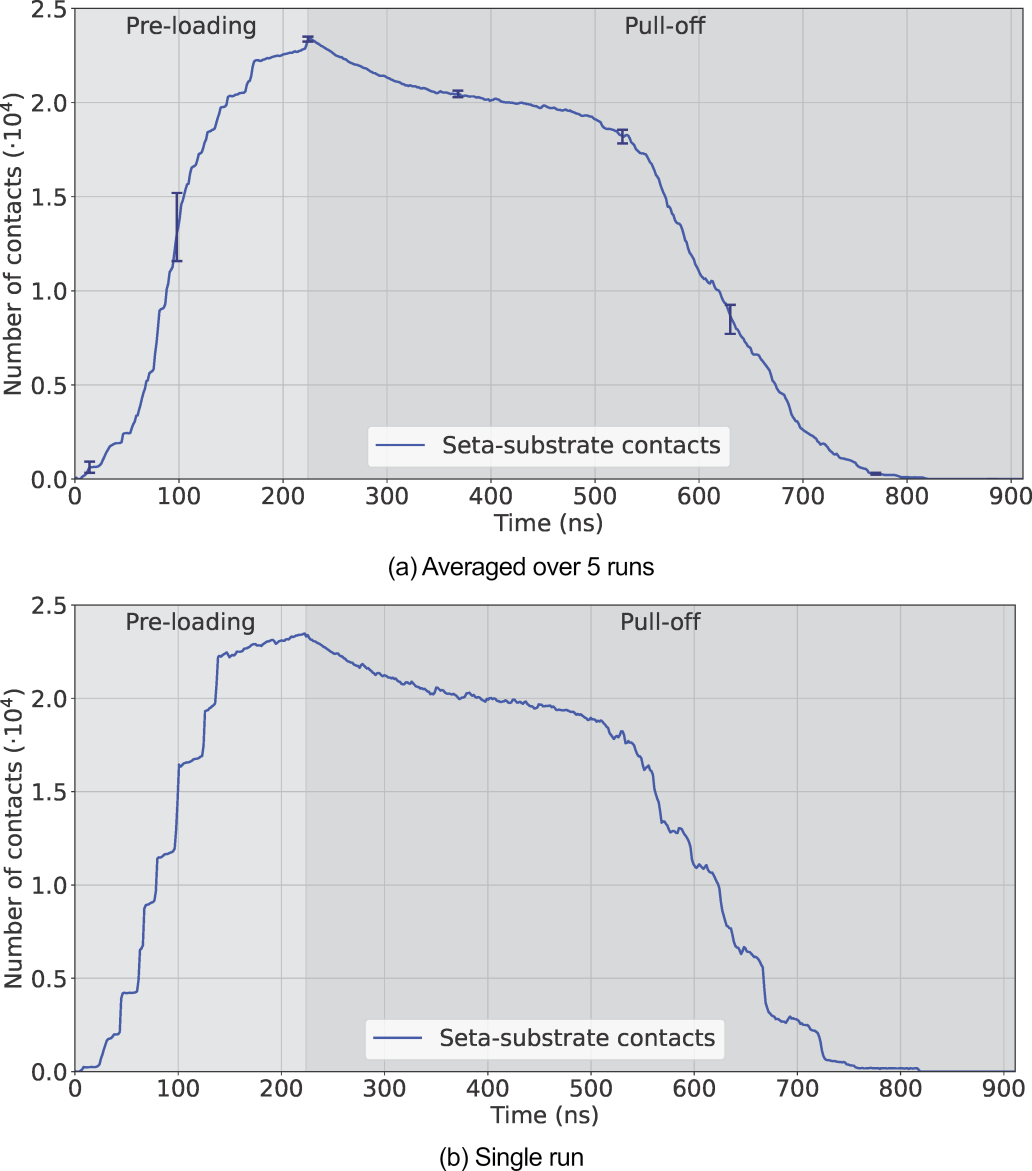
Number of seta–substrate contacts. (a) Averaged over five runs with error bars in a darker blue color at interesting points. (b) For a single run.

The contact profile in Figure 9a was calculated by summing the number of contact points over all spatulae and then averaging over the five runs, while Figure 9b is the contact profile for a single run. They follow a similar trend to the seta–substrate force profiles (Figure 8). During preloading, contacts increased as spatulae were pressed against the substrate. In the pull-off phase, the number of contact points decreased gradually as the seta retracted, until individual spatulae started to detach completely (at ≈526 ns). This is marked by the sudden increase in the rate of contact loss (negative slope). Eventually, all spatulae completely detached from the substrate. Furthermore, the curve for a single run (Figure 9b) shows discrete steps when the final few spatulae detach one by one between 500 and 850 ns. Animations of the entire multiscale seta are available in Supporting Information File 2.

### Spatula behavior

**Figure 10:**
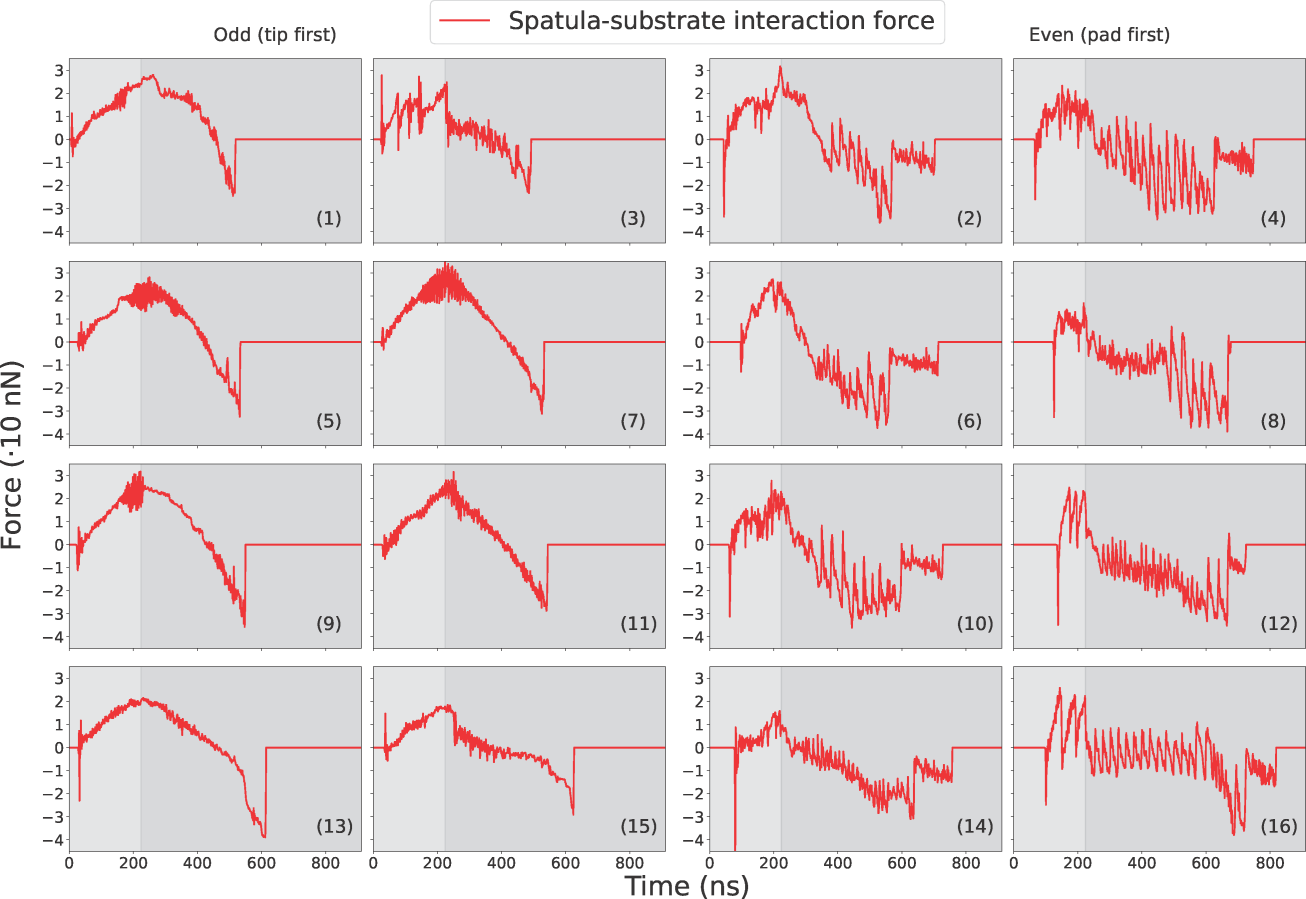
Spatula–substrate force profiles for every spatula for a typical pull-off run. For numbering of spatulae, see Figure 2. Sign convention as in Figure 8: positive = compression, negative = adhesion.

**Figure 11:**
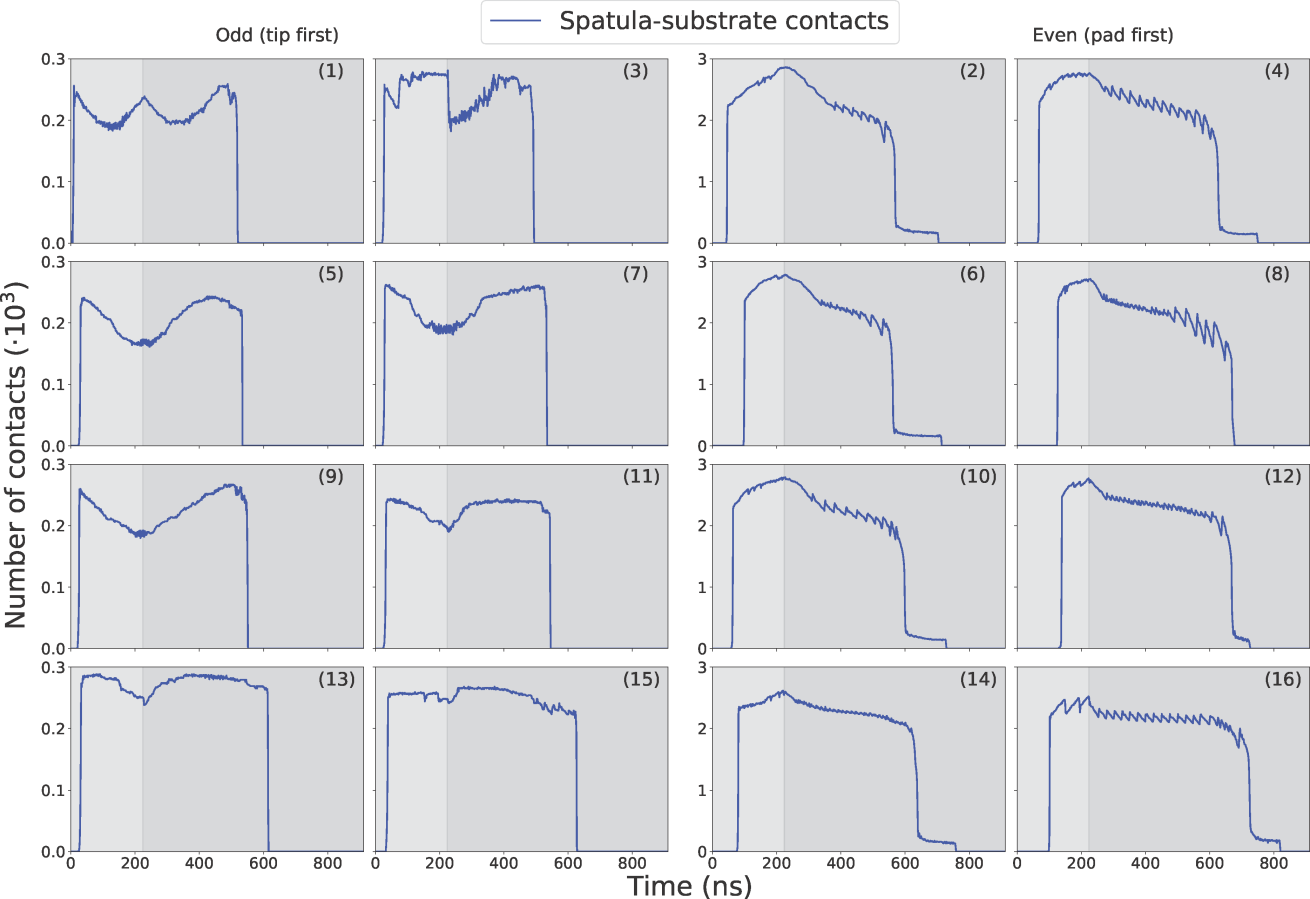
Spatula-substrate contact profiles of every spatula for a typical pull-off run. For numbering of spatulae, see Figure 2

**Figure 12:**
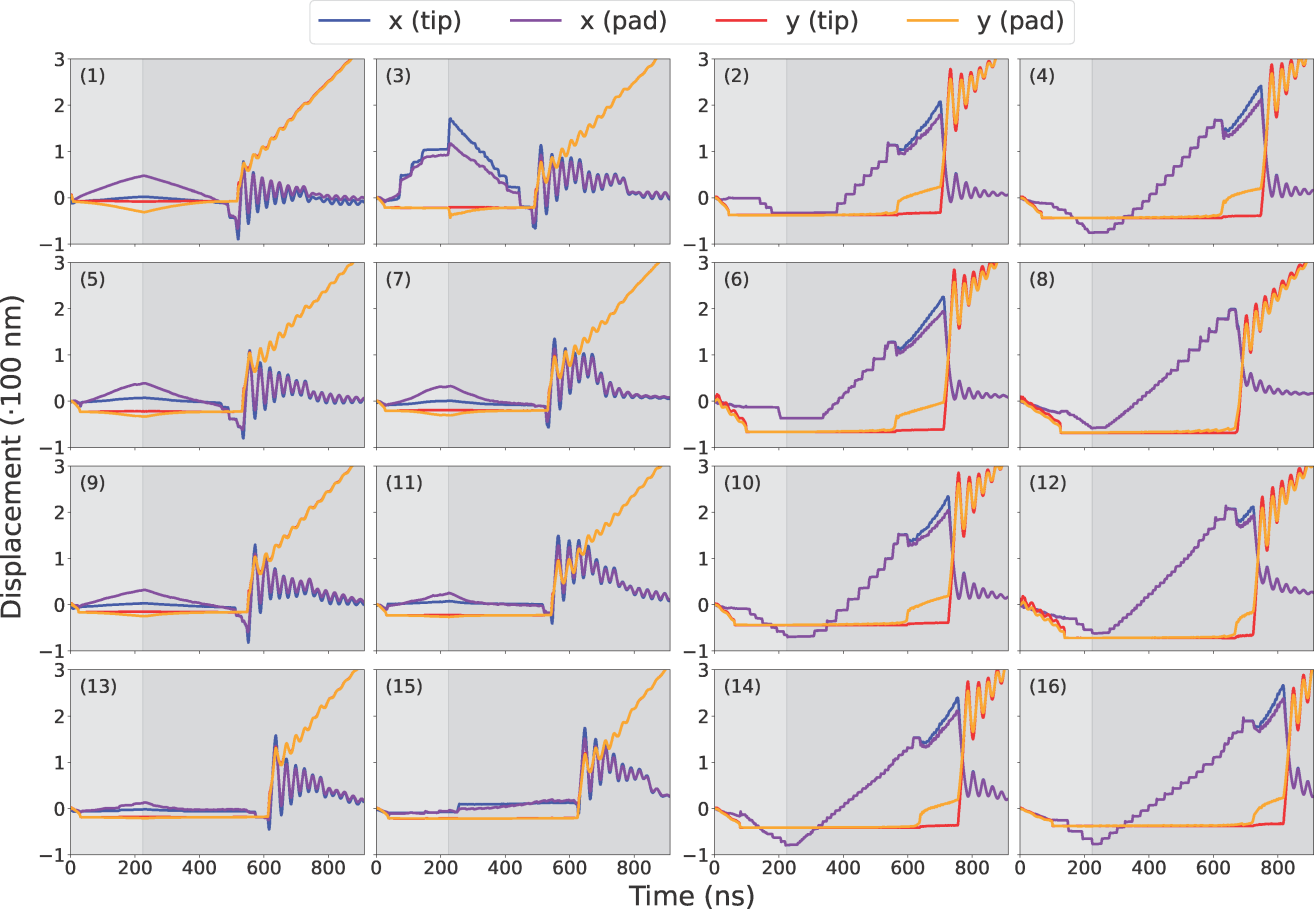
Spatula tip and pad displacement profiles along *X*-axis (along the surface in the direction of seta inclination) and *Y*-axis (surface normal) of every spatula for a typical pull-off run. For numbering of spatulae, see Figure 2; for the designation of tip and pad beads, see Figure 5.

Since all spatulae share the same coarse-grained spatula–substrate potential, differences in adhesion originate from structure and geometry rather than interface chemistry. We analyzed the 16 spatulae individually. Figure 10, Figure 11, and Figure 12 report, respectively, their force, contact, and displacement profiles in a representative run. Across runs, orientation (pad- vs tip-dominant contact), branch position along the hierarchy (Figure 2), and transient shear/sliding modulate the peak adhesion force (|*F*_min_|) and detachment order (Table 4, below).

#### Pre-loading phase

**Figure 13:**
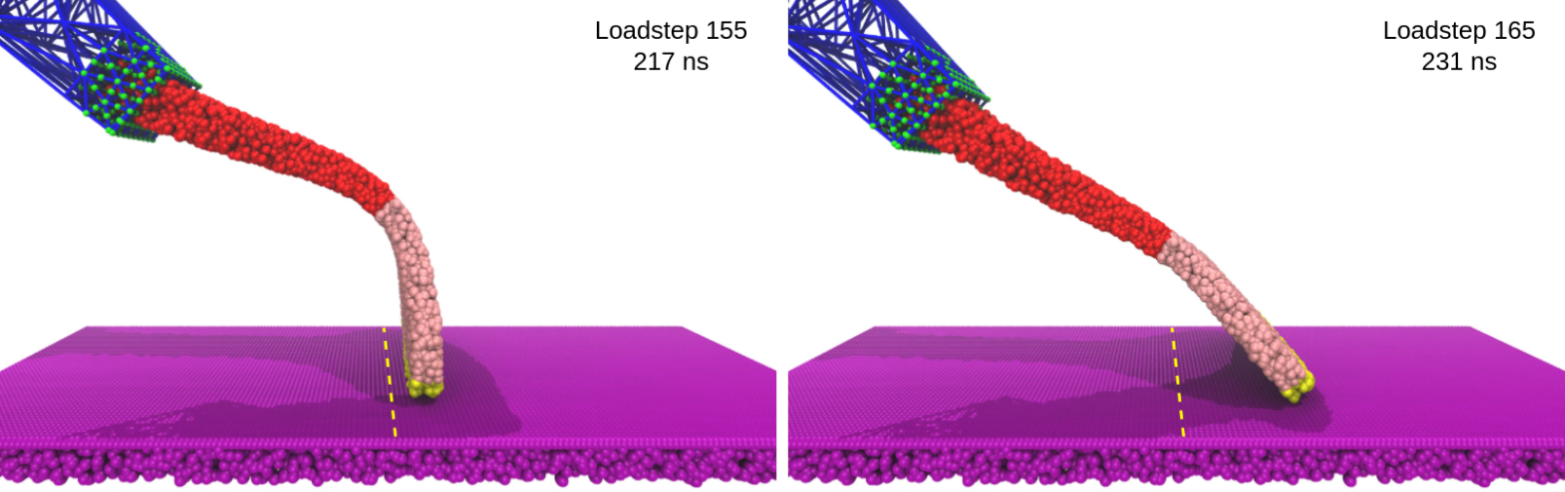
Snapshots of spatula 3 just before (load step 155; 217 ns) and after (load step 165; 231 ns) a tip slip event that occurred at the end of the preloading phase (load step 160; 224 ns). Yellow beads = spatula tip, pink beads = spatula pad, red beads = spatula shaft, green = anchor points, and blue = FE mesh. The yellow dashed line on the substrate is a marker near the initial spatula contact point.

As the spatulae were pressed against the substrate, the compressive force increased (positive in Figure 10). The contact and displacement profiles provide additional insights: Odd-numbered spatulae (1,3,5,…,15) approached the substrate tip-first. Upon initial contact, their contact number increased, but as loading continued, the spatula bent slightly, causing the tip to tilt. This reduced the number of contacts as only the edge of the tip remained in contact. In terms of displacement, the tip’s *X*- and *Y*-coordinates remained nearly unchanged, while the pad’s *X*-coordinate increased slightly due to bending. The extent of bending decreased progressively from spatula 1 to 16, as shown by the increasing *X*-displacement of the pad while the tip displacement remained close to zero in Figure 12. In some cases, such as spatula 1, the tip tilted so far that parts of the pad came into contact with the substrate, causing an increase in the contact number (Figure 11). Spatula 3 exhibited a different behavior; its tip slid during preload and slipped at the end of it, which is evident in the tip displacement profile (Figure 12). Here, we use the term “sliding” to refer to gradual lateral movement of the spatula tip or pad across the substrate, and “slipping” to describe a sudden loss of contact followed by lateral motion. The contact profile for spatula 3 shows a zig-zag pattern, initial contact loss from tip tilting, followed by recovery from sliding and re-tilting, and finally a sharp drop due to tip slippage. Figure 13 shows snapshots of spatula 3 just before and after this tip slip event. Full animations of all spatulae are available in Supporting Information File 3. In contrast, Even-numbered spatulae (2,4,6,…,16) approached the substrate with their pads nearly parallel, leading to a classic jump-to-contact (snap-in in AFM terminology) upon first entering the attractive range. This transition from no-contact to intimate contact is visible in the animations in Supporting Information File 3. This resulted in a sharp, immediate increase in contact number, approximately ten times higher than that of odd-numbered spatulae, and a sharp negative spike in the adhesion force as seen in Figure 11 and Figure 10, respectively. Further loading gradually increased the number of contacts as more spatula beads came within the substrate’s interaction range. The displacement profiles in Figure 12 show that, during preload, both the tip and pad *X*-coordinates start decreasing at a certain point, indicating sliding toward the *−X*-direction. Higher-numbered spatulae initiated sliding earlier and underwent greater displacement, indicating that sliding became more pronounced farther from the pulling point.

**Figure 14:**
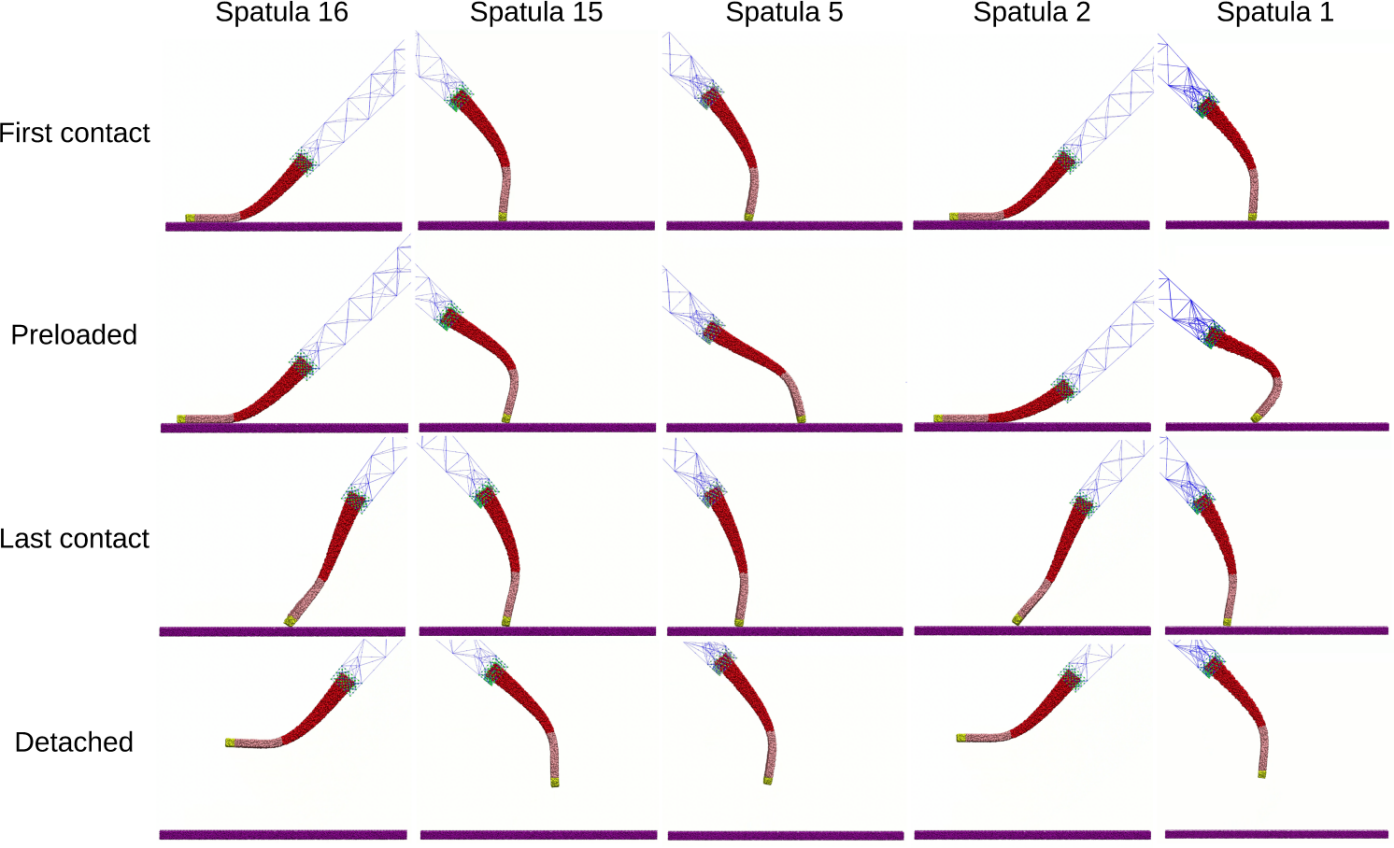
Snapshots of different spatulae at interesting stages during pull-off simulations. Yellow = spatula tip, pink = spatula pad, red = spatula shaft, green = anchor points, and blue = FE mesh.

This behavior is clearly visible for a few example spatulae in the first two rows of Figure 14, that is, the moment of first contact, and the end of the preloading phase. The animations of individual spatulae provided in Supporting Information File 3 show these behaviors with more clarity. Figure 14 shows that odd-numbered spatulae maintained contact primarily through their tips, while even-numbered spatulae achieved full pad contact without requiring shearing.

#### Pull-off phase

During pull-off, spatulae experienced bending, shearing, and detachment, depending on their orientation and distance from the pulling point: As pull-off began, odd-numbered spatulae (tip-dominated contact) unbent (Figure 12), restoring the tip to a more perpendicular orientation. This resulted in a temporary increase in contact number before reaching a plateau. The compressive force gradually decreased, reaching zero before transitioning to a tensile (adhesive) force (negative in Figure 10). This force increased in magnitude until complete detachment occurred. Just before detachment, the tip’s and pad’s *X*-coordinates decreased slightly, suggesting a minor slide towards *−X* before the spatula completely detached. At detachment, there was a sudden increase in the tip’s *Y*-coordinate, marking separation from the substrate. After detachment, the spatula exhibited post-detachment oscillations, visible in fluctuations of its *Y*-coordinate, due to the release of stored elastic energy. The most negative force (peak adhesion) occurred just before the spatula tip fully detached from the substrate. Averaged across all tip-dominant spatulae, this adhesion magnitude was 30.5 ± 4.9 nN. This behavior is visible in the third row of Figure 14. It shows the moment of last contact for odd-numbered spatulae when the tip adhered to the substrate just before final detachment. In contrast, even-numbered spatulae (pad-dominated contact) underwent peeling and shearing simultaneously. Initially, as pull-off began, the tip’s and pad’s *Y*-coordinates remained nearly unchanged, but their *X*-coordinates increased, indicating sliding in *+X*, as the spatula moved back towards its original position. Just before detachment, the pad lost contact with the substrate, causing a sudden increase in its *Y*-coordinate, while the tip remained adhered to the substrate. The tip continued to slide in *X* even after the pad lifted, meaning that the spatula detached partially, while the tip remained in contact and underwent additional sliding. Finally, once the tip also detached, both the tip and pad moved away together, with post-detachment oscillations due to stored elastic energy being released. For these spatulae, the most negative (peak adhesion) force typically occurred earlier, specifically, when the pad detached from the substrate. After this, the spatula continued to adhere via its tip, but the corresponding force magnitudes were lower. The average adhesion magnitude across all pad-dominant spatulae was 35.9 ± 2.3 nN. The third row of Figure 14 illustrates the moment of last contact for even-numbered spatulae, when the pad had detached but the tip remained in contact just before final detachment. This two-stage detachment mechanism, where the pad peels off first, followed by the tip, is clearly visible in the contact profile, where the number of contacts sharply dropped to a non-zero plateau before eventually dropping to zero.

The animations in Supporting Information File 2 provide a clear visualization of the detachment sequence and mechanisms of individual spatulae. For the run used in our Figures Figure 10–Figure 14 and in the discussion so far, Table 4 lists the spatula detachment time in the order of detachment. The table confirms that the odd-numbered spatulae detached before even-numbered ones and that spatulae closer to the pulling force (i.e., lower-numbered) detached earlier.

**Table 4:** Load steps at which spatulae detached in the order of detachment for a typical pull-off run.

Spatula no.	Detachment load step	Detachment time (ns)

3	352	492.8
1	371	519.4
7	381	533.4
5	382	534.8
11	389	544.6
9	393	550.2
13	439	614.6
15	448	627.2
8	484	677.6
2	502	702.8
6	510	714.0
12	518	725.2
10	520	728.0
4	535	749.0
14	541	757.4
16	585	819.0

#### Sliding and friction

Since our force field was never intended to reproduce macroscopic friction forces (see Subsection ”Spatulae and substrate: particles”), and our substrate model is exceedingly smooth compared to real surfaces, agreement with experimental friction values at comparable sliding velocities was not a goal of this study. Nonetheless, our simulations revealed significant spatula sliding, particularly among the pad-dominant (even-numbered) spatulae (Figure 2). During the 400–600 ns time interval, as visible in the pad displacement profiles (Figure 12), the in-plane displacement (*X*) of both the tips and pads increased nearly linearly, while the normal position (*Y*) remained constant. This indicates a sliding regime during which the contact numbers steadily decreased (Figure 11) and the adhesive forces increased (became more negative) (Figure 10). We estimated the sliding velocity in this regime by averaging the slopes of the pad displacements of all even-numbered (pad-dominant) spatulae, arriving at 0.66 ± 0.11 m/s. By contrast, shear speeds in AFM experiments range from 0.5 μm/s to 0.1 m/s [8–9,60], meaning our simulated shear speeds exceeded even the fastest experimental rates several times. This is probably due to a much higher loading rate and a defect-free, smooth substrate in the simulations.

The friction coefficient μ is the unitless ratio of frictional force to normal force. Friction coefficients in gecko adhesion studies vary widely depending on anatomical scale. At the whole-foot or whole-toe level, shear-supported adhesion often yields μ values between 1 and 10, depending on the shear speed [9,60]. However, experiments using isolated gecko setal arrays, more comparable in scale to our model, report values in the range of 0.3–0.7 on smooth glass substrates at low shear speeds (0.1 mm/s) [60–61]. We conducted supplementary pure MD simulations of individual pad-dominant spatula–substrate systems subjected to preloading (up to 20 nN) followed by shear at 0.1, 1, 10, and 100 m/s. These simulations displaced the APs in the normal direction for preload, and then in the *+X* direction for shear, while constraining the other two dimensions. Each simulation was repeated three times with different spatula–substrate systems. At the highest shear speed (100 m/s), the spatula deformed unphysically rather than sliding. At the lower speeds, however, we observed clear sliding and recorded both adhesion and lateral forces to compute friction coefficients. These were 0.30 ± 0.05, 0.19 ± 0.02, and 0.16 ± 0.01 for shear speeds of 0.1, 1, and 10 m/s, respectively. This downward trend is consistent with experimental observations, where the friction coefficient is known to decrease with increasing shear velocity.

In the seta pull-off simulations, the apparent friction coefficient (averaged over forty instances, i.e., five runs times eight pad-dominant spatulae) was 0.55 ± 0.13, likely elevated compared to the pure MD shear simulations because sliding was overlayed with peel-off. Notably, this value falls within the 0.3–0.7 range measured for isolated setal arrays, despite our simulated shear velocity (0.66 m/s) being orders of magnitude higher than in those experiments.

### Comparison to literature

Our seta geometry and loading process were designed to closely follow the pure finite element model of a branched seta developed by Sauer and colleagues [21]. As with their model, ours represents an idealization of the natural geometry of gecko setae [57,62]. Sauer et al.’s simulations demonstrated how the peeling of spatulae upon retraction could extend the distance over which adhesion forces act before detachment, effectively increasing the “range” of adhesion. Their model also captured the sequential detachment of spatulae, a feature consistent with experimental observations [58]. However, their reported spatula pull-off force of 7.9 nN was not a predicted outcome of the simulation, but a prescribed input. In their contact formulation, the adhesive force was applied at a single point and defined by user input. In contrast, our model calculated the pull-off force as an emergent result of molecular interactions distributed over the entire spatula pad. This distinction between localized point contact and distributed area contact led to differences in detachment behavior and force response. In their model, detachment was instantaneous, with no further interaction following separation. In our simulations, detachment proceeded gradually via peeling, and interactions continued as long as some spatula beads remained within the non-bonded interaction range (12 nm) of the substrate. Their seta model used a Young’s modulus of 2 GPa and Poisson’s ratio of 0.2, values that were selected without direct experimental validation. These parameters resulted in a more compliant seta capable of greater deformation, which increased adhesion range but reduced the peak pull-off force. Our material parameters were ultimately derived from atomistic keratin simulations [12], which resulted in a stiffer response. The mesoscale spatula geometry in our model was based on SEM images from Xu and colleagues [23]. Our spatula was modeled using coarse-grained particles, whereas theirs used finite elements. Sauer et al. simulated spatula–substrate adhesion through a Lennard-Jones potential parameterized in earlier work [29]. Although their framework allowed for the inclusion of tangential sticking forces, this aspect was not explored in detail. Our model also used a potential (see Subsection ”Spatulae and substrate: particles”) parameterized against pull-off pressures from atomistic simulations. Like them, we did not explicitly account for frictional forces, and our substrate was atomically smooth, which affected sliding behavior. Additionally, their simulations are equilibrium in nature, while we perform non-equilibrium simulations with high loading rates. Consequently, our computed average spatula peak pull-off force was higher, at 33.2 ± 2.6 nN.

Our simulation approach followed the general protocol used in atomic force microscopy (AFM) experiments, particularly those by Huber et al. [58] and Niederegger and colleagues [63]. Our computed spatula peak pull-off force (≈33 nN) was higher than the 8–10 nN reported by Huber et al., and the 4–20 nN range observed in humidity-controlled studies by Huber et al. and Sun and colleagues [35,57,59]. Note that our geometry led to perfect contact of an ideal spatula with an ideal substrate, whereas in experiment, roughnesses and defects on both sides will lower the effective adhesion. Despite these systematic differences, our results fell within the broader range of experimental observations and captured behaviors such as bending, sliding, and peeling.

## Conclusion

This paper presented a coupled multiscale molecular dynamics (MD) and finite element method (FEM) technique for simulating gecko seta adhesion on substrates. By adopting a hybrid approach, we avoided key limitations of purely MD-based or purely FEM-based simulations. Our study built on the seta geometry of Sauer et al. [21–22], as well as on multiscale MD–FEM simulations in polymer research, where they have been shown to be successful [43,47–48,50].

At the core of our work was a gecko seta-spatula model that integrated mesoscale molecular dynamics for the spatula–substrate interface with finite element calculations for the larger, micrometer-scale seta. It employed a spatula–substrate force field derived from atomistic (united-atom) simulations of gecko keratin. Even with an idealized seta geometry and the linear-elastic FE model, the multiscale simulations revealed a wider array of detachment mechanisms, including peeling, sliding, and bending. The study underscored how relatively small changes in spatula orientation could significantly alter load distribution and contact evolution. The results aligned with experimental AFM observations and prior computational findings, reproducing pull-off forces within the reported ranges. Additionally, the model captured mechanisms, such as spatula bending and sliding on the substrate, behaviors not permitted in earlier computational models and still inaccessible to current experimental methods. Furthermore, the results demonstrated that the MD–FEM non-equilibrium coupling scheme could reproduce key features and force magnitudes consistent with pure non-equilibrium MD simulations. Animations illustrating these behaviors are available in the Supporting Information File 1. Taken together, our results indicate that, at fixed interface chemistry, the realized pull-off is governed by geometry and load sharing through the branched seta, features captured by the concurrent MD–FEM coupling.

In future work, further refinements could incorporate more realistic substrates and seta structures, including hierarchical branching, rough substrates, or chemical patterning, ultimately bringing simulations closer to biological reality. We therefore view this concurrent MD–FE method as a stepping stone for more comprehensive studies of gecko adhesion, as well as an adaptable platform for exploring other bioadhesive phenomena.

## Supporting Information

File 1 Discussion around the effects and choice of parameters.

File 2 Animations of the entire multiscale seta and a zoomed version focusing on all 16 spatulae.

File 3 16 Animations focusing on individual spatulae; adjacent spatulae have been omitted for clarity.

## Data Availability

Simulation files, scripts, and code to reproduce all results from this paper are available at: https://tudatalib.ulb.tu-darmstadt.de/handle/tudatalib/4618
