# Supplementary material for: Molecular and mechanical insights into gecko seta adhesion: multiscale simulations combining molecular dynamics and the finite element method
Source: Beilstein J Nanotechnol. 2025 Nov 14;16:2055–76. doi: 10.3762/bjnano.16.141 (PMC12621623; doi:10.3762/bjnano.16.141)
Supplement: File 1 — Discussion around the effects and choice of parameters. [file Beilstein_J_Nanotechnol-16-2055-s001.pdf]

BEILSTEIN JOURNAL OF NANOTECHNOLOGY

Supporting Information

for

Molecular and mechanical insights into gecko seta adhesion: multiscale simulations combining molecular dynamics and the finite element method

Yash Jain, Saeed Norouzi, Tobias Materzok, Stanislav N. Gorb and Florian Müller-Plathe

Beilstein J. Nanotechnol. **2025,** *16,* 2055–2076. doi:10.3762/bjnano.16.141

Discussion around the effects and choice of parameters

License and Terms: This is a supporting information file under the terms of the Creative Commons Attribution License (https://creativecommons.org/licenses/by/4.0). Please note that the reuse, redistribution and reproduction in particular requires that the author(s) and source are credited and that individual graphics may be subject to special legal provisions.
The license is subject to the *Beilstein Journal of Nanotechnology* terms and conditions: (https://www.beilstein-journals.org/bjnano/terms)

Effect of the FE-coupling Spring Constant

As described in Section “Controlling FEM deformation” of the main text, the FE-coupling spring constant k_{FE} connects finite element (FE) nodes in the bridging domain (BD) to their initial positions during each FEM iteration. This harmonic restraint acts as a penalty term that counters the constant forces applied to the bridging domain FE nodes (i.e., the averaged anchor point forces from MD) and prevents excessive mesh deformation. Conceptually, k_{FE} governs the “step size” that the BD is allowed to take towards equilibrium position in each iteration. Meanwhile, the number of FE-MD iterations per load step determines how many such steps the system takes before applying the next load increment. The interplay of these two parameters controls how well the molecular (MD) and continuum (FE) domains remain synchronized during both pre-loading and pull-off.

To isolate the effect of k_{FE}, we performed simulations with 0.5×, 1×, 2×, 4×, and 100× the chosen production value of 0.08 nN/nm, while keeping the number of iterations per load step fixed to 10. Figure S1 illustrates the resulting force profiles for the substrate (blue), anchor points (red), and FE driver nodes (green). As in the main text, compression/loading is taken as positive and adhesion during pull-off as negative. When we refer to an “adhesion peak,” this corresponds to the *minimum* of the force curve; reported adhesion values are given as magnitudes, $|F_{\text{min}}|$. At very high stiffness (100×), the bridging domain becomes overly rigid: the BD FE nodes (which share positions with the corresponding APs in the MD domain) cannot move enough to accommodate local spatula-substrate interactions, leading to unrealistic compression or stretching of the FE mesh. In contrast, reducing k_{FE} to 0.5× makes the domain too compliant, allowing large displacements in the BD and inducing instabilities in the MD domain. Intermediate values (2×, 4×) show partial improvements in force agreement but still exhibit noticeable deviations. The baseline case (1×) achieves a practical balance, with the three forces aligned across most of the simulation.

Effect of the number of FE-MD iterations per loadstep

Varying the iteration count with fixed loadstep duration

With k_{FE} fixed to the baseline production value, we studied how the number of FE-MD iterations per loadstep influences the force profiles. Simulations were carried out with 1, 4, 7, and 10 iterations per loadstep, while keeping the total simulation time per loadstep Δt^{ls} fixed at 1.4 ns to preserve the overall loading rate of 1.88×10^{12} pN/s. Consequently, the time allocated for the MD portion in each iteration, Δt^{iter}, varies inversely with the number of iterations per loadstep δn^{iter} (e.g., 1.4 ns for 1 iteration vs. 0.14 ns for 10 iterations), as described by equation 1 in the main text.

At the end of each MD segment, anchor point (AP) forces are averaged over the final 50% of the MD trajectory and passed to the FE solver for the next iteration. Figure S2 shows the resulting force profiles for the substrate (blue), anchor points (red), and FE driver nodes (green) across the different iteration counts. With only 1 iteration per loadstep, the FE force curve diverges considerably from the other two, particularly in the pull-off (adhesive, negative) regime and around detachment. As the number of iterations increases, the three force profiles come into progressively better agreement, indicating improved synchronization between the domains. At 10 iterations, the curves remain closely aligned throughout most of the simulation. However, some mismatch persists in the post-detachment regime, where rapid motion and residual elastic oscillations make force convergence more difficult.

Each additional FE-MD iteration per loadstep increases the computational cost, and beyond 10 iterations per loadstep, our full-system simulations became impractical on the hardware used. Thus, 10 iterations represents a practical compromise between domain synchronisation and computational feasibility.

Extended Iteration Tests at Key Loadsteps

Even with 10 iterations per loadstep, divergence among the three force curves (FE, AP, and substrate) often reappears during later stages of pull-off—especially near and after complete detachment. To examine this more closely, we selected four representative loadsteps from a production simulation: end of preloading (LS160), mid pull-off (LS260), near peak adhesion (most negative force; LS376),

and post-detachment (LS450). At each of these loadsteps, we performed up to 400 additional FE-MD iterations *without advancing the load*, allowing the system to progressively equilibrate to the applied deformation. Each iteration consisted of 200 ps of MD simulation.

Resulting Figure S3 shows that the number of iterations required for force convergence increases noticeably with loadstep index. LS160 converges relatively quickly, while LS376 and LS450—corresponding to peak adhesion and post-detachment—require hundreds of iterations for the FE, AP, and substrate forces to align. This explains the divergence observed in Figures S1 and S2, even at 10 iterations per loadstep: later loadsteps involve faster, more nonlinear dynamics and therefore require significantly more iterations to reach equilibrium.

For proper convergence in a rate-dependent simulation — particularly during later stages — both the number of iterations and the FE-coupling spring constant must be carefully tuned to match the dynamic behavior of the system. Our choice of 10 iterations per loadstep and the baseline k_{FE} represents a practical compromise between accuracy and computational feasibility on the HPC resources used.

A more adaptive solution could involve defining a formal *a priori* convergence criterion based on the rate of change in AP forces or energy, enabling the algorithm to dynamically determine the number of FE-MD iterations required for each loadstep. Of course, the total simulation time per loadstep Δt^{ls} would need to remain fixed to maintain a consistent loading rate.

Alternatively, if quasi-static behavior is desired, one could increase both the time per iteration Δt^{iter} and the iteration count δn^{iter}, thereby increasing Δt^{ls} accordingly. However, this would also substantially increase the computational cost of the simulation.

Spatula animations

Animations from one of our production simulations are also included in the Supporting Information. These animations illustrate the behavior of the multi-scale seta system during pull-off. A total of 18 movies are provided, categorized as follows:

- **Full multiscale seta pull-off:**

An overview animation showing the entire multiscale model, including the FE mesh and all 16 MD spatulae, from the start to the end of the pull-off.

File name: `seta_full.mp4`

- **Multiscale seta focusing on the 16 spatulae:** A closer view highlighting all the spatulae and their concurrent interactions with the substrate.

  File name: `seta_zoom.mp4`

- **Individual spatula zoom-ins (16 total):**

  Separate animations focused on each of the 16 spatulae. Neighboring spatulae are hidden to provide a clear, unobstructed view of individual bending, peeling, sliding, and detachment behaviors. The spatula pad beads are colored pink, the tip beads are yellow, while the rest of the molecular spatula shaft is composed of red colored beads.

  File names: `spatula_1.mp4`, `spatula_2.mp4`, . . . , `spatula_16.mp4`

These animations collectively illustrate the key mechanisms discussed in the main text, including bending, peeling, sliding, and tip-detachment across various stages of the multiscale pull-off simulation.

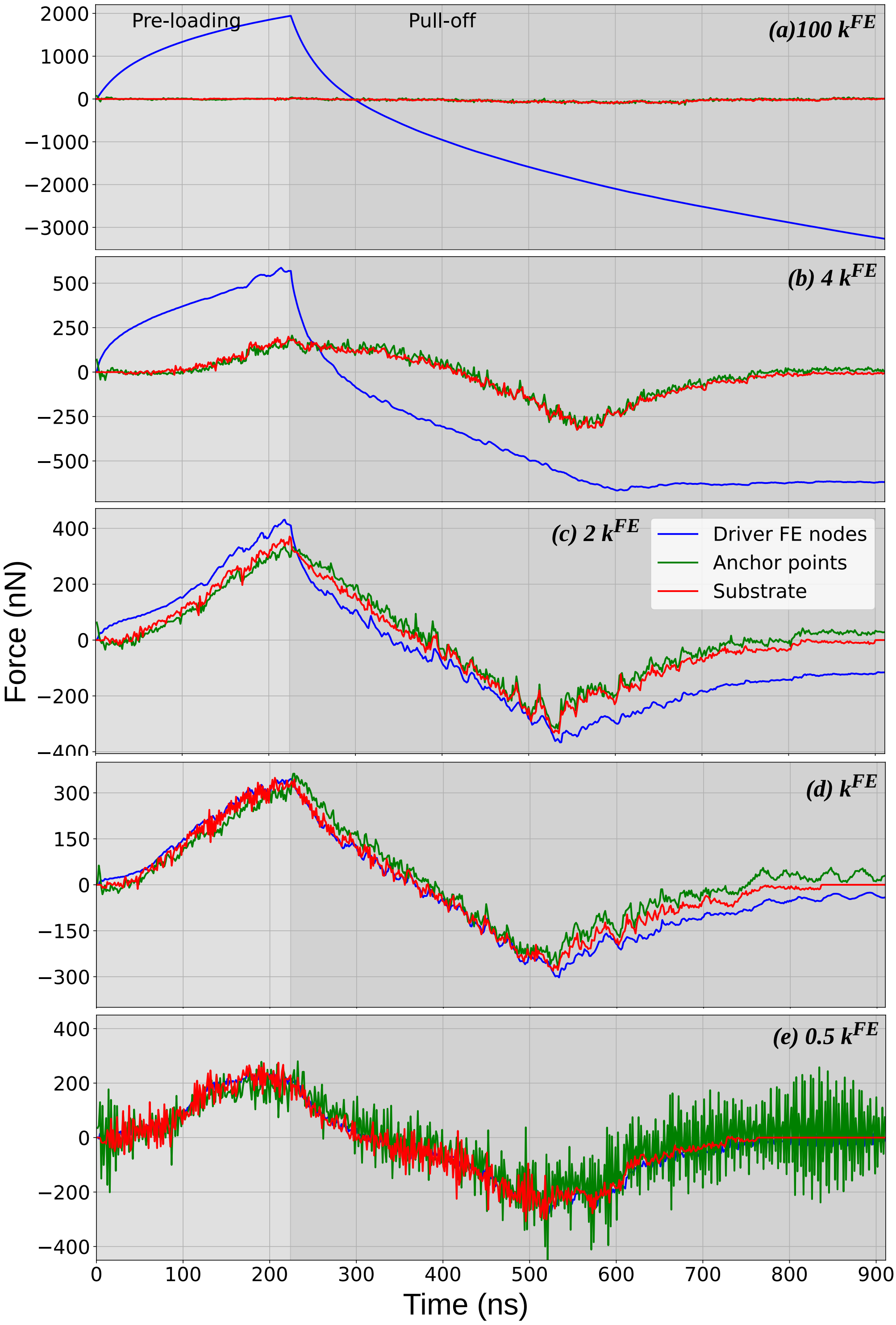

Figure S1: Force profiles under different FE-coupling spring constants. Substrate (blue), anchor points (red), and FE driver nodes (green) forces are shown for 0.5×, 1×, 2×, 4×, and 100× the chosen k_{FE}. Overly large stiffness (100×) produces unrealistic compression/stretching, while overly small stiffness (0.5×) leads to large BD displacements and fluctuations. The baseline (1×) yields stable alignment among all three force profiles.

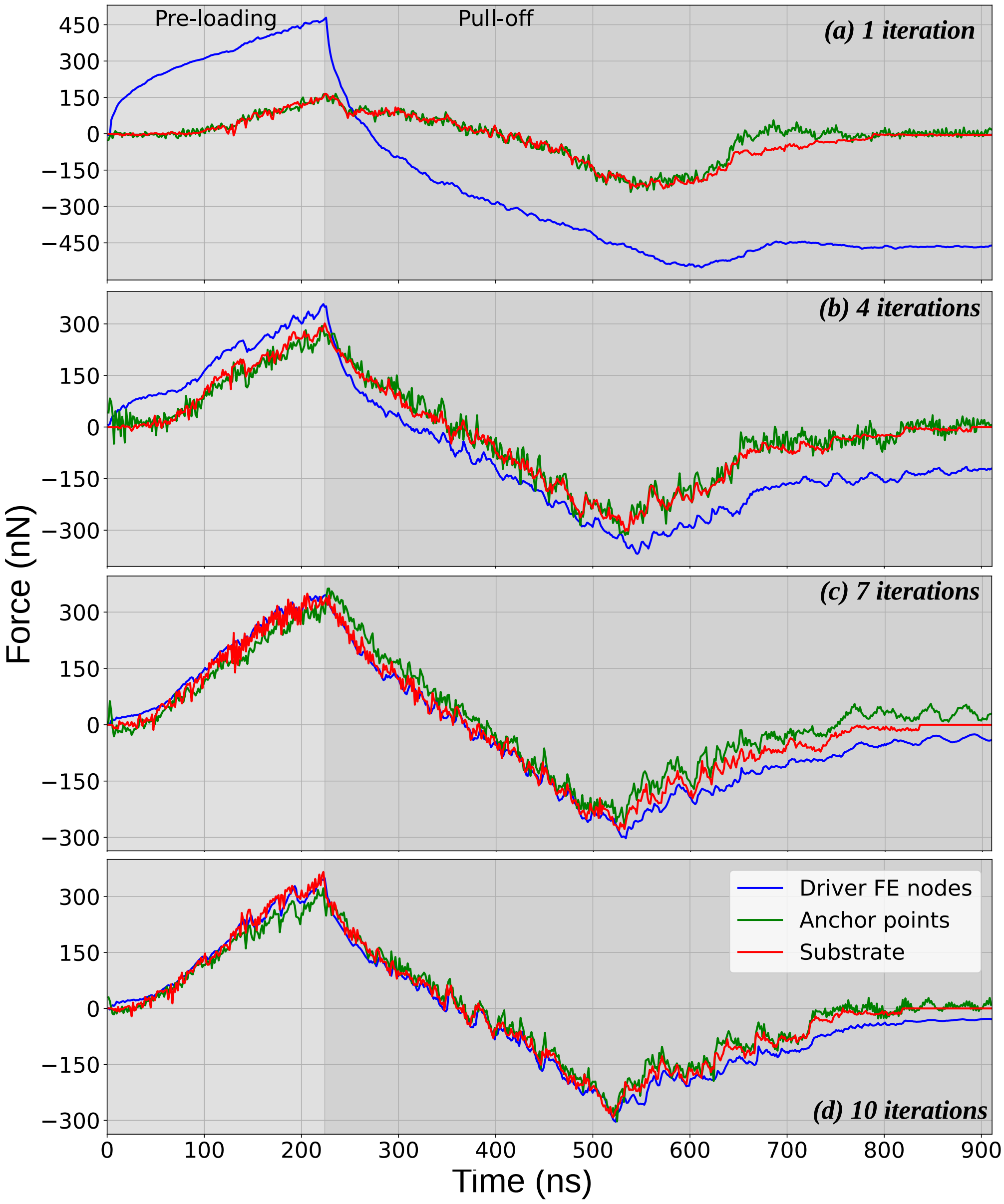

Figure S2: Force profiles comparing substrate (blue), anchor points (red), and FE driver nodes (green) for different FE-MD iteration counts (1, 4, 7, 10) per loadstep. The total MD time per loadstep is fixed at 1.4 ns. Higher iteration counts improve force alignment, but none achieve full alignment in later stages.

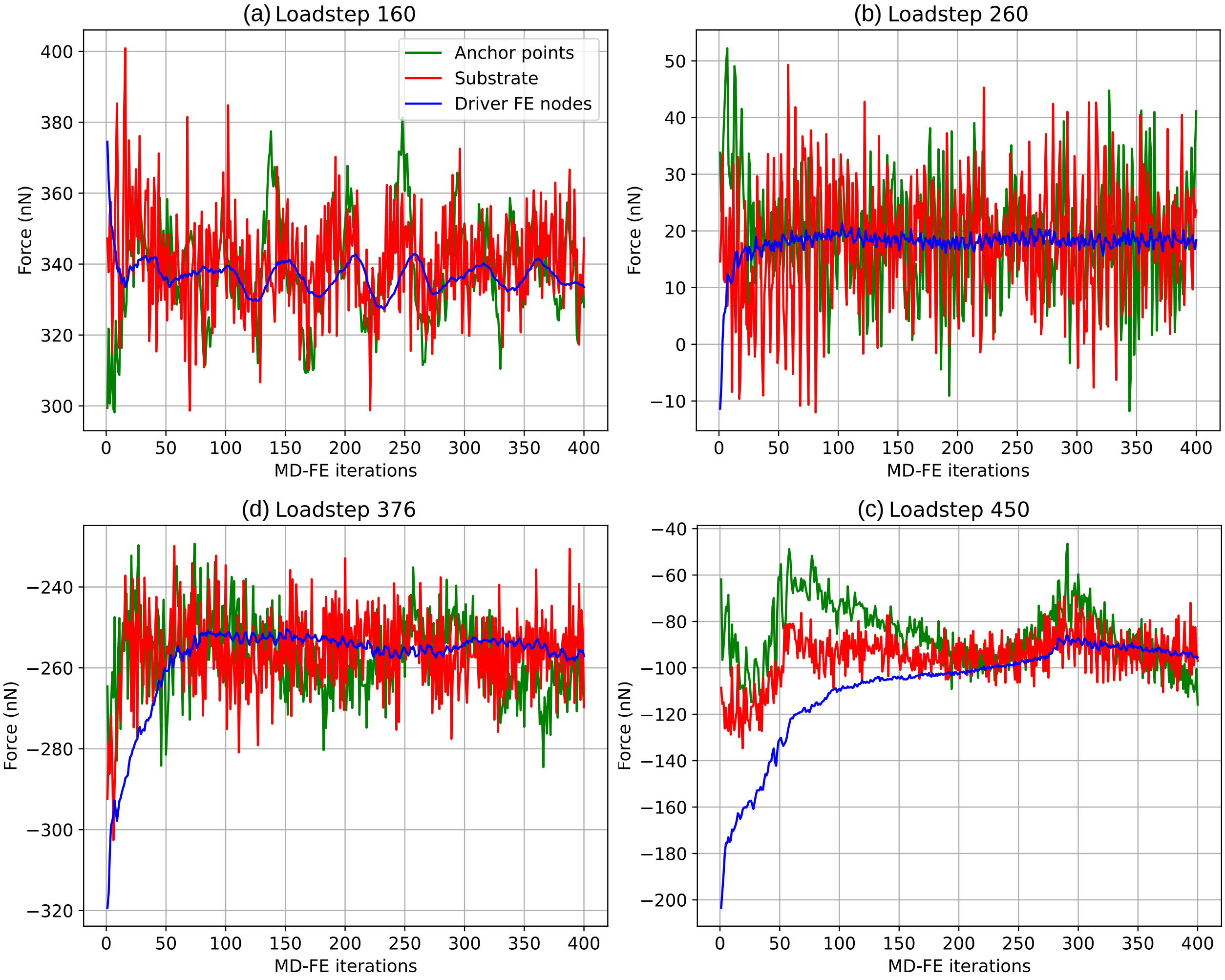

Figure S3: Extended iteration tests at LS160, LS260, LS376, and LS450, each extended up to 400 FE-MD iterations with 200 ps of MD time per iteration. The required iteration count to achieve near-convergence grows significantly in later loadsteps.
